# Changes in ambient environmental conditions correspond to variations in *in vitro* fertilization outcomes during the COVID-19 pandemic

**DOI:** 10.3389/fendo.2026.1796050

**Published:** 2026-06-09

**Authors:** Heeyon Kim, Hyun Jun Kim, Soyeong Park, Yun Soo Chung, Jin Kyung Baek, Yebon Kim, Jeongmi Yoon, Chungsoon Ryu, Bo Hyon Yun, Young Sik Choi, Daewoo Pak, Seok Kyo Seo, Yohan Ko

**Affiliations:** 1 Department of Obstetrics and Gynecology, Severance Hospital, Yonsei University College of Medicine, Seoul, Republic of Korea; 2 Institute of Women’s Life Medical Science, Yonsei University College of Medicine, Seoul, Republic of Korea; 3 Department of Computer Science, Yonsei University, Wonju, Republic of Korea; 4 Department of Medical Information, Yonsei Wonju Health System, Wonju, Republic of Korea; 5 Department of Computing, Imperial College London, London, United Kingdom; 6 Division of Data Science, Yonsei University, Wonju, Republic of Korea; 7 Division of Software, Yonsei University, Wonju, Republic of Korea

**Keywords:** air pollution, COVID-19, fertilization rate, *in vitro* fertilization, oocyte maturation

## Abstract

**Background:**

Air pollution is associated with impaired oocyte and embryo development, but the modifying effects of large-scale societal disruptions remain unclear. The aim of this study was to examine whether *in vitro* fertilization (IVF)-related mature oocyte, fertilization, and pregnancy rates differ across the periods before, during, and after the coronavirus disease 2019 (COVID-19) pandemic. We further evaluated whether ambient environmental exposures modified these differences.

**Methods:**

We retrospectively analyzed oocyte retrieval cycles performed at a single tertiary infertility center in Seoul, South Korea (2018–2024). Complete-case analyses included 1, 871 cycles for mature oocyte rate, 1, 234 cycles for fertilization rate, and 662 cycles for clinical pregnancy. Environmental exposures (PM_2_._5_, PM_10_, NO_2_, O_3_, CO, SO_2_, temperature, humidity) were assessed over follicular-phase, 90-day, and 365-day windows. Beta regression (mature oocyte and fertilization rates) and logistic regression (pregnancy) models were fitted with cluster-robust standard errors, adjusting for maternal age, AMH (except pregnancy), treatment indication, controlled ovarian stimulation protocol, fertilization method, and total motile sperm count.

**Results:**

The mature oocyte rate was significantly lower in the during-COVID (β = −0.32, 95% CI −0.45 to −0.19, p < 0.001) and post-COVID (β = −0.45, 95% CI −0.59 to −0.31, p < 0.001) groups than in the pre-COVID reference. The fertilization rate was higher in the post-COVID group than in the pre-COVID group in the regression model (β = 0.22, 95% CI 0.02 to 0.42, p = 0.030), whereas the during-COVID comparison was not significant (p = 0.637). Pregnancy rate did not differ significantly across the three periods (Wald χ² = 2.12, p = 0.346). Long-term (365-day) exposures exhibited significant Env × COVID interactions on the Likelihood Ratio Test for mature oocyte rate (O_3_: LRT p = 0.002; CO: LRT p = 0.008; NO_2_: LRT p = 0.009). No environmental interactions were significant for fertilization rate. For clinical pregnancy, five Env × COVID interactions reached nominal significance, including SO_2_ × 90-day (LRT p = 0.014), temperature × 365-day (p = 0.013), and O_3_ × 365-day (p = 0.020).

**Conclusions:**

IVF outcomes varied across COVID-19 pandemic periods, and long-term ambient environmental exposures modified these effects, particularly for oocyte maturation. Recent and long-term environmental exposures should be considered when evaluating IVF success.

## 1 Introduction

Infertility affects approximately 15% of couples worldwide and represents a substantial global health challenge, prompting intensive research on modifiable risk factors (1–3). *In vitro* fertilization (IVF) is a cornerstone of infertility and reproductive medicine, with over 2.5 million cycles performed annually worldwide (4). IVF success is critically dependent on three sequential biological processes: oocyte maturation, fertilization, and embryo implantation; each process is influenced by intrinsic patient factors and extrinsic environmental conditions (5, 6). Female factor infertility accounts for approximately 40%–50% of all infertility cases. Older age at first childbearing, along with increasing environmental exposures, have raised concerns regarding female reproductive health in industrialized nations (7, 8).

Among various environmental determinants, ambient air pollution has emerged as a critical factor specifically influencing female reproductive health outcomes through multiple pathophysiological mechanisms (9, 10). Exposure to particulate matter (PM_2.5_, PM_10_), nitrogen dioxide (NO_2_), ozone (O_3_), and other gaseous pollutants can induce oxidative stress, systemic inflammation, and endocrine disruption, resulting in impaired folliculogenesis, reduced oocyte quality, and dysregulated embryo development (11–18). Considering the multiple stages of female reproduction, assessing pollutant exposure over several biologically relevant time windows is crucial for identifying the long-term, intermediate, and acute environmental effects on oocyte and embryo development and implantation.

The coronavirus disease 2019 (COVID-19) pandemic, which was declared in March 2020, has created unprecedented global changes in societal, behavioral, and environmental conditions. Although stringent lockdowns, reduced vehicular traffic, and decreased industrial activity led to air quality improvement, with reductions in NO_2_, carbon monoxide (CO), sulfur dioxide (SO_2_), and particulate matter of 20%–50% in urban areas, O_3_ levels increased owing to altered atmospheric chemistry (19–21). Concurrently, psychological stress due to social isolation, economic uncertainty, health concerns, and changes in physical activity and lifestyle alter neuroendocrine pathways and reproductive processes (22–24). Seoul, the capital of South Korea, offers a suitable context for investigating these complex dynamics because targeted intervention strategies, such as intensive testing, contact tracing, and localized restrictions, rather than complete lockdowns, enabled relatively stable healthcare access throughout the pandemic period (25, 26). Additionally, its distinct seasonal climate patterns produce substantial variability in temperature, humidity, and air pollutant levels, which are useful for environmental exposure analyses (27).

Existing literature primarily focuses on comparing specific pollutant concentrations with IVF outcomes or analyzing the direct effect of single time windows, neglecting long-term exposure and contextual factors such as a pandemic period. Moreover, the influence of ambient environmental variations on large-scale societal disruptions that affect gamete development, fertilization, and early implantation success remains unclear.

Therefore, the present study aimed to determine whether IVF outcomes, including mature oocyte, fertilization, and clinical pregnancy rates, differed across distinct pandemic phases. We further evaluated how ambient air pollution and meteorological factors modified IVF outcomes, and whether their effects differed according to the COVID-19 pandemic period through interaction analyses. We hypothesized that the pandemic-related changes resulted in a measurable interaction effect, reflecting altered biological and behavioral responses to environmental exposure.

## 2 Materials and methods

### 2.1 Study population

This retrospective cohort study of 2, 162 oocyte retrieval cycles was conducted at Severance Hospital, a single tertiary medical center in Seoul, South Korea, from January 2018 to December 2024. The term oocyte retrieval cycle is used rather than IVF cycle because the cohort comprises indications for (1) infertility treatment with the intent to proceed with IVF or intracytoplasmic sperm injection (ICSI), (2) oocyte or embryo cryopreservation before anticipated cancer treatment, and (3) elective oocyte cryopreservation. For each cycle, the index date was the oocyte pickup (OPU) date. Donor-gamete cycles, frozen embryo transfer cycles, and cycles with premature discontinuation were excluded from the study. The primary dataset comprised all OPU cycles for the mature oocyte rate (number of metaphase II [MII] oocytes or total retrieved oocytes, n = 2162). Cycles that proceeded to insemination (IVF or ICSI) were included in the fertilization rate analysis (number of fertilized oocytes or number of MII oocytes; n = 1234), and cycles with embryo transfer were included in the clinical pregnancy analysis (ultrasound-confirmed intrauterine gestational sac per transfer; n = 662). These analytic samples (1, 871 cycles from 1, 101 patients for the mature oocyte rate analysis, 1, 234 cycles from 678 patients for the fertilization rate analysis, and 662 cycles from 418 patients for the pregnancy analysis) were used throughout the study; **Table 1** describes the total cohort (N = 2, 162) while **Tables 2**–**4**: **Supplementary Table 1** present the analytic results. Complete-case analysis was applied, excluding cycles with missing values in any required covariates. Controlled ovarian stimulation (COS) and transvaginal oocyte retrieval were performed according to standardized institutional protocols. All cycles with ≥ 1 oocyte retrieved were eligible for the study.

**Table 1:** Descriptive characteristics of OPU outcomes across the COVID-19 periods (N = 2162).

Variable	Total (N = 2162)	Pre-COVID (N = 469)	During-COVID (N = 951)	Post-COVID (N = 742)	p-value
Age (years)	35.92 ± 5.85	34.49 ± 6.24	36.00 ± 5.55	36.71 ± 5.81	< 0.001***
AMH (ng/mL)	1.83 ± 2.19	2.01 ± 2.14	1.79 ± 2.12	1.77 ± 2.29	0.012*
Mature oocyte ratio (%)	72.9 ± 27.6	79.6 ± 25.5	71.7 ± 28.5	70.1 ± 26.9	< 0.001***
Fertilization rate (%)	76.5 ± 27.6	74.0 ± 30.2	74.5 ± 27.6	80.3 ± 26.2	< 0.001***
Pregnancy outcome					0.132
No pregnancy	430 (64.8%)	120 (68.2%)	204 (62.4%)	106 (65.8%)	
Biochemical pregnancy	54 (8.1%)	10 (5.7%)	25 (7.6%)	19 (11.8%)	
Intrauterine pregnancy	180 (27.1%)	46 (26.1%)	98 (30.0%)	36 (22.4%)	

Data are expressed as mean ± standard deviation or n (%).

*p < 0.05, ***p < 0.001.

**Table 2:** Direct effects of the COVID-19 pandemic on IVF outcomes.

A. Wald tests for COVID-19 period effects																			
Reference	Comparison			Mature oocyte rate					Fertilization rate						Pregnancy rate				
				χ²		df	p		χ²		df		p		χ²		df	p	
Pre-COVID	During-COVID			21.39		1	< 0.001***		0.41		1		1.000		0.51		1	1.000	
Pre-COVID	Post-COVID			41.34		1	< 0.001***		4.74		1		0.089		0.35		1	1.000	
During-COVID	Post-COVID			3.63		1	0.171		7.62		1		0.017*		2.02		1	0.465	
Overall	(df = 2)			43.41		2	< 0.001***		8.98		2		0.015*		2.12		2	0.346	

B. Beta regression coefficients																			
Variable		Mature oocyte rate						Fertilization rate						Pregnancy rate					
		β (95% CI)	OR		p			β (95% CI)		OR		p		β (95% CI)		OR			p
Intercept		1.18*** (0.76, 1.61)	3.26		< 0.001***			1.21*** (0.64, 1.78)		3.34		< 0.001***		4.32*** (2.63, 6.02)		75.33			< 0.001***
During-COVID		-0.32*** (-0.45, -0.19)	0.73		< 0.001***			0.05 (-0.14, 0.24)		1.05		0.637		0.17 (-0.31, 0.65)		1.19			0.477
Post-COVID		-0.45*** (-0.59, -0.31)	0.64		< 0.001***			0.22* (0.02, 0.42)		1.25		0.030*		-0.17 (-0.74, 0.40)		0.84			0.555
Age		-0.00 (-0.01, 0.01)	1.00		0.545			-0.01 (-0.02, 0.01)		0.99		0.468		-0.15*** (-0.20, -0.10)		0.86			< 0.001***
AMH		-0.05*** (-0.08, -0.02)	0.95		< 0.001***			-0.04** (-0.07, -0.01)		0.96		0.004**		—		—			—
Indication: Fertility preservation		-0.04 (-0.20, 0.12)	0.96		0.589			-0.15 (-0.33, 0.03)		0.86		0.107		0.10 (-0.86, 1.06)		1.11			0.834
Indication: Social freezing		0.34*** (0.21, 0.47)	1.4		< 0.001***			-0.71*** (-0.85, -0.56)		0.49		< 0.001***		—		—			—
Indication: PGT		0.03 (-0.23, 0.29)	1.03		0.803			-0.06 (-0.34, 0.22)		0.94		0.681		-14.26*** (-15.54, -12.97)		0.0			< 0.001***
Protocol: Soft		0.26*** (0.14, 0.39)	1.3		< 0.001***			0.17* (0.01, 0.33)		1.19		0.034*		0.20 (-0.30, 0.70)		1.22			0.429
Protocol: Long agonist		-0.01 (-0.32, 0.31)	0.99		0.966			0.16 (-0.13, 0.45)		1.17		0.282		-0.61 (-1.61, 0.40)		0.54			0.235
Protocol: Microflare		0.02 (-0.21, 0.25)	1.02		0.868			0.17 (-0.02, 0.36)		1.18		0.088		-0.37 (-0.96, 0.22)		0.69			0.217
Fertilization: all IVF		—	—		—			-0.75*** (-1.08, -0.43)		0.47		< 0.001***		0.52 (-0.22, 1.27)		1.69			0.168
Fertilization: IVF+ICSI		—	—		—			-0.37*** (-0.50, -0.24)		0.69		< 0.001***		0.47* (0.06, 0.88)		1.6			0.026*
TMSC		—	—		—			0.00** (0.00, 0.00)		1.0		0.003**		-0.00 (-0.00, 0.00)		1.0			0.426

Pairwise p-values Bonferroni-corrected (×3 within each outcome); overall Wald tests (df = 2) are uncorrected.

*p < 0.05, ***p < 0.001.

AMH, anti-Müllerian hormone; OR, odds ratio, CI, 95% confidence interval; COVID-19, coronavirus disease 2019; OPU, oocyte pickup; PGT, preimplantation genetic testing; IVF, in vitro fertilization; ICSI, intracytoplasmic sperm injection; TMSC, total motile sperm count. Reference categories: Pre-COVID (COVID period); Infertility (indication); GnRH antagonist (protocol); all ICSI (fertilization method). TMSC coefficients are shown in scientific notation due to small per-unit effect size. AMH was not applicable to the pregnancy model.

**Table 3:** Interactions between environmental exposures and COVID-19 periods on mature oocyte rates.

A. Beta regression: p-values for model selection																																								
Variable			Period					N			Environment				Age					AMH				COVID				Env × COVID					Indication					Protocol		
CO			Period 1					1,871			0.584				0.544					< 0.001***				< 0.001***				0.128					< 0.001***					< 0.001***		
Humidity											0.305				0.552					< 0.001***				< 0.001***				0.927					< 0.001***					< 0.001***		
NO_2_											0.824				0.545					< 0.001***				< 0.001***				0.401					< 0.001***					< 0.001***		
O_3_											0.596				0.55					< 0.001***				< 0.001***				0.284					< 0.001***					< 0.001***		
PM_10_											0.198				0.551					< 0.001***				< 0.001***				0.086					< 0.001***					< 0.001***		
PM_2.5_											0.76				0.542					< 0.001***				< 0.001***				0.382					< 0.001***					< 0.001***		
SO_2_											0.825				0.551					< 0.001***				0.001**				0.460					< 0.001***					< 0.001***		
Temperature											0.801				0.544					< 0.001***				< 0.001***				0.502					< 0.001***					< 0.001***		
CO			90 days								0.511				0.534					< 0.001***				< 0.001***				0.450					< 0.001***					< 0.001***		
Humidity											0.316				0.538					< 0.001***				< 0.001***				0.574					< 0.001***					0.001**		
NO_2_											0.481				0.533					< 0.001***				< 0.001***				0.819					< 0.001***					< 0.001***		
O_3_											0.643				0.54					< 0.001***				< 0.001***				0.165					< 0.001***					< 0.001***		
PM_10_											0.327				0.538					< 0.001***				< 0.001***				0.509					< 0.001***					0.001**		
PM_2.5_											0.391				0.529					< 0.001***				< 0.001***				0.738					< 0.001***					0.001**		
SO_2_											0.487				0.52					< 0.001***				< 0.001***				0.237					< 0.001***					< 0.001***		
Temperature											0.259				0.533					< 0.001***				< 0.001***				0.851					< 0.001***					< 0.001***		
CO			365 days								0.267				0.561					< 0.001***				0.130				0.008**					< 0.001***					< 0.001***		
Humidity											0.453				0.575					< 0.001***				0.173				0.146					< 0.001***					< 0.001***		
NO_2_											0.386				0.578					< 0.001***				0.396				0.009**					< 0.001***					< 0.001***		
O_3_											0.419				0.573					< 0.001***				0.012*				0.002**					< 0.001***					< 0.001***		
PM_10_											0.518				0.556					< 0.001***				0.003**				0.129					< 0.001***					< 0.001***		
PM_2.5_											0.421				0.57					< 0.001***				0.148				0.062					< 0.001***					< 0.001***		
SO_2_											0.591				0.569					< 0.001***				0.222				0.047*					< 0.001***					< 0.001***		
Temperature											0.333				0.527					< 0.001***				< 0.001***				0.295					< 0.001***					< 0.001***		

B. All interactions between environmental exposures and COVID-19 periods on mature oocyte rate (N = 1871)																																								
Variable		Period			Intercept		Environment			During			Post				Env × During		Env × Post			Age			AMH		Fert. preservation			Social freezing		PGT			Soft		Long agonist		Microflare	
CO		Period 1			1.24*** (0.81, 1.67)		-0.10* (-0.20, -0.01)			-0.37*** (-0.51, -0.22)			-0.50*** (-0.65, -0.35)				0.15* (0.01, 0.28)		0.15** (0.04, 0.27)			-0.00 (-0.01, 0.01)			-0.05*** (-0.08, -0.02)		-0.04 (-0.20, 0.12)			0.34*** (0.21, 0.47)		0.04 (-0.22, 0.29)			0.27*** (0.14, 0.39)		-0.02 (-0.33, 0.29)		0.02 (-0.20, 0.25)	
Humidity					1.21*** (0.78, 1.65)		0.05 (-0.08, 0.18)			-0.36*** (-0.52, -0.19)			-0.50*** (-0.67, -0.33)				-0.03 (-0.18, 0.12)		-0.01 (-0.17, 0.15)			-0.00 (-0.01, 0.01)			-0.05*** (-0.08, -0.02)		-0.04 (-0.20, 0.12)			0.34*** (0.21, 0.47)		0.03 (-0.23, 0.30)			0.26*** (0.14, 0.39)		-0.01 (-0.32, 0.31)		0.02 (-0.20, 0.25)	
NO_2_					1.23*** (0.79, 1.67)		-0.05 (-0.15, 0.05)			-0.36*** (-0.54, -0.19)			-0.51*** (-0.70, -0.32)				0.11 (-0.02, 0.24)		0.04 (-0.11, 0.18)			-0.00 (-0.01, 0.01)			-0.05*** (-0.08, -0.02)		-0.05 (-0.21, 0.11)			0.34*** (0.21, 0.47)		0.03 (-0.23, 0.29)			0.27*** (0.14, 0.39)		0.00 (-0.32, 0.32)		0.02 (-0.20, 0.25)	
O_3_					1.22*** (0.79, 1.65)		0.09 (-0.02, 0.20)			-0.37*** (-0.52, -0.22)			-0.49*** (-0.65, -0.34)				-0.13 (-0.27, 0.01)		-0.12 (-0.27, 0.02)			-0.00 (-0.01, 0.01)			-0.05*** (-0.08, -0.02)		-0.04 (-0.20, 0.12)			0.34*** (0.21, 0.47)		0.02 (-0.24, 0.28)			0.26*** (0.14, 0.39)		-0.01 (-0.33, 0.30)		0.03 (-0.20, 0.26)	
PM_10_					1.21*** (0.78, 1.64)		-0.11* (-0.19, -0.02)			-0.35*** (-0.49, -0.21)			-0.50*** (-0.64, -0.35)				0.15* (0.03, 0.27)		0.02 (-0.10, 0.15)			-0.00 (-0.01, 0.01)			-0.05*** (-0.08, -0.02)		-0.05 (-0.21, 0.11)			0.34*** (0.21, 0.47)		0.02 (-0.24, 0.28)			0.27*** (0.14, 0.40)		0.02 (-0.30, 0.34)		0.02 (-0.21, 0.24)	
PM_2.5_					1.22*** (0.79, 1.65)		-0.06 (-0.13, 0.00)			-0.35*** (-0.49, -0.20)			-0.49*** (-0.64, -0.34)				0.10 (-0.01, 0.21)		0.05 (-0.06, 0.16)			-0.00 (-0.01, 0.01)			-0.05*** (-0.08, -0.02)		-0.05 (-0.21, 0.11)			0.34*** (0.21, 0.48)		0.03 (-0.23, 0.29)			0.27*** (0.14, 0.39)		0.00 (-0.31, 0.32)		0.01 (-0.21, 0.24)	
SO_2_					1.22*** (0.76, 1.68)		-0.02 (-0.12, 0.07)			-0.33** (-0.54, -0.13)			-0.51*** (-0.75, -0.28)				0.11 (-0.06, 0.27)		-0.03 (-0.22, 0.17)			-0.00 (-0.01, 0.01)			-0.05*** (-0.08, -0.02)		-0.05 (-0.21, 0.11)			0.34*** (0.21, 0.47)		0.02 (-0.24, 0.28)			0.27*** (0.14, 0.39)		0.00 (-0.32, 0.32)		0.02 (-0.21, 0.24)	
Temperature					1.19*** (0.76, 1.61)		0.07 (-0.03, 0.17)			-0.33*** (-0.47, -0.20)			-0.47*** (-0.61, -0.33)				-0.08 (-0.20, 0.05)		-0.08 (-0.22, 0.05)			-0.00 (-0.01, 0.01)			-0.05*** (-0.08, -0.02)		-0.04 (-0.20, 0.12)			0.34*** (0.21, 0.47)		0.03 (-0.23, 0.29)			0.27*** (0.14, 0.39)		-0.01 (-0.33, 0.31)		0.02 (-0.20, 0.25)	
CO		90 days			1.23*** (0.80, 1.66)		-0.08 (-0.18, 0.01)			-0.38*** (-0.53, -0.23)			-0.50*** (-0.65, -0.35)				0.06 (-0.07, 0.20)		0.10 (-0.03, 0.23)			-0.00 (-0.01, 0.01)			-0.05*** (-0.08, -0.02)		-0.04 (-0.20, 0.12)			0.34*** (0.21, 0.48)		0.04 (-0.22, 0.30)			0.26*** (0.14, 0.39)		-0.02 (-0.34, 0.30)		0.02 (-0.21, 0.25)	
Humidity					1.16*** (0.72, 1.61)		-0.02 (-0.16, 0.12)			-0.30** (-0.49, -0.11)			-0.47*** (-0.67, -0.27)				0.04 (-0.12, 0.21)		0.10 (-0.07, 0.28)			-0.00 (-0.01, 0.01)			-0.05*** (-0.08, -0.02)		-0.04 (-0.20, 0.12)			0.33*** (0.20, 0.46)		0.03 (-0.23, 0.29)			0.26*** (0.13, 0.39)		-0.00 (-0.32, 0.31)		0.03 (-0.20, 0.26)	
NO_2_					1.23*** (0.78, 1.67)		-0.04 (-0.16, 0.08)			-0.36*** (-0.55, -0.17)			-0.52*** (-0.73, -0.32)				0.04 (-0.12, 0.20)		-0.01 (-0.18, 0.15)			-0.00 (-0.01, 0.01)			-0.05*** (-0.08, -0.02)		-0.04 (-0.20, 0.12)			0.34*** (0.21, 0.47)		0.03 (-0.23, 0.29)			0.26*** (0.14, 0.39)		-0.00 (-0.32, 0.32)		0.02 (-0.21, 0.25)	
O_3_					1.26*** (0.83, 1.69)		0.14* (0.02, 0.25)			-0.41*** (-0.56, -0.26)			-0.54*** (-0.70, -0.38)				-0.16* (-0.31, -0.02)		-0.13 (-0.29, 0.02)			-0.00 (-0.01, 0.01)			-0.05*** (-0.08, -0.02)		-0.04 (-0.20, 0.12)			0.34*** (0.21, 0.47)		0.03 (-0.23, 0.29)			0.27*** (0.14, 0.39)		-0.00 (-0.32, 0.31)		0.02 (-0.20, 0.25)	
PM_10_					1.19*** (0.76, 1.61)		-0.00 (-0.09, 0.09)			-0.32*** (-0.46, -0.18)			-0.47*** (-0.62, -0.32)				-0.00 (-0.14, 0.13)		-0.07 (-0.20, 0.06)			-0.00 (-0.01, 0.01)			-0.05*** (-0.08, -0.02)		-0.04 (-0.20, 0.12)			0.33*** (0.20, 0.46)		0.02 (-0.24, 0.28)			0.26*** (0.13, 0.39)		-0.00 (-0.32, 0.32)		0.02 (-0.20, 0.25)	
PM_2.5_					1.19*** (0.76, 1.62)		-0.00 (-0.08, 0.08)			-0.32*** (-0.47, -0.17)			-0.47*** (-0.63, -0.32)				-0.02 (-0.15, 0.11)		-0.06 (-0.20, 0.07)			-0.00 (-0.01, 0.01)			-0.05*** (-0.08, -0.02)		-0.04 (-0.20, 0.12)			0.33*** (0.20, 0.46)		0.03 (-0.24, 0.29)			0.26*** (0.13, 0.39)		-0.00 (-0.32, 0.31)		0.03 (-0.20, 0.25)	
SO_2_					1.26*** (0.81, 1.72)		-0.05 (-0.15, 0.06)			-0.38*** (-0.59, -0.16)			-0.68*** (-0.95, -0.41)				0.10 (-0.10, 0.31)		-0.20 (-0.47, 0.08)			-0.00 (-0.01, 0.01)			-0.05*** (-0.08, -0.02)		-0.04 (-0.20, 0.12)			0.33*** (0.20, 0.46)		0.01 (-0.25, 0.27)			0.26*** (0.14, 0.39)		0.01 (-0.31, 0.33)		0.02 (-0.21, 0.24)	
Temperature					1.19*** (0.77, 1.62)		0.06 (-0.03, 0.16)			-0.33*** (-0.47, -0.20)			-0.47*** (-0.61, -0.33)				-0.04 (-0.17, 0.09)		-0.04 (-0.17, 0.09)			-0.00 (-0.01, 0.01)			-0.05*** (-0.08, -0.02)		-0.04 (-0.20, 0.12)			0.34*** (0.21, 0.47)		0.03 (-0.23, 0.30)			0.26*** (0.14, 0.39)		-0.01 (-0.33, 0.31)		0.02 (-0.20, 0.25)	
CO		365 days			1.25*** (0.79, 1.71)		-0.06 (-0.19, 0.07)			-0.36** (-0.59, -0.14)			-0.25 (-0.58, 0.08)				0.36** (0.09, 0.62)		0.46* (0.08, 0.83)			-0.00 (-0.01, 0.01)			-0.05*** (-0.08, -0.02)		-0.04 (-0.20, 0.12)			0.34*** (0.21, 0.47)		-0.04 (-0.30, 0.22)			0.27*** (0.14, 0.40)		-0.01 (-0.33, 0.31)		-0.01 (-0.25, 0.22)	
Humidity					1.21*** (0.68, 1.73)		0.03 (-0.15, 0.20)			-0.34* (-0.66, -0.02)			-0.66** (-1.08, -0.25)				-0.17 (-0.43, 0.08)		0.18 (-0.17, 0.53)			-0.00 (-0.01, 0.01)			-0.05*** (-0.08, -0.02)		-0.04 (-0.20, 0.12)			0.34*** (0.21, 0.47)		-0.03 (-0.29, 0.23)			0.27*** (0.14, 0.39)		-0.01 (-0.33, 0.31)		0.00 (-0.23, 0.24)	
NO_2_					1.27*** (0.73, 1.82)		-0.07 (-0.27, 0.14)			-0.41* (-0.76, -0.05)			-0.88** (-1.45, -0.32)				0.42* (0.08, 0.75)		-0.28 (-0.76, 0.21)			-0.00 (-0.01, 0.01)			-0.05*** (-0.08, -0.02)		-0.04 (-0.20, 0.12)			0.34*** (0.21, 0.47)		-0.05 (-0.31, 0.21)			0.27*** (0.14, 0.39)		-0.01 (-0.32, 0.31)		-0.01 (-0.24, 0.22)	
O_3_					1.28*** (0.81, 1.74)		0.09 (-0.06, 0.25)			-0.47*** (-0.70, -0.24)			-0.76*** (-1.12, -0.40)				-0.33** (-0.55, -0.11)		0.10 (-0.21, 0.41)			-0.00 (-0.01, 0.01)			-0.05*** (-0.08, -0.02)		-0.04 (-0.20, 0.12)			0.34*** (0.21, 0.47)		-0.06 (-0.33, 0.21)			0.27*** (0.14, 0.40)		-0.02 (-0.35, 0.30)		-0.01 (-0.24, 0.22)	
PM_10_					1.21*** (0.76, 1.67)		-0.02 (-0.13, 0.09)			-0.28** (-0.50, -0.07)			-0.49*** (-0.73, -0.26)				0.23* (0.01, 0.44)		0.01 (-0.20, 0.21)			-0.00 (-0.01, 0.01)			-0.05*** (-0.08, -0.02)		-0.05 (-0.21, 0.11)			0.34*** (0.21, 0.47)		0.03 (-0.23, 0.29)			0.26*** (0.14, 0.39)		-0.00 (-0.32, 0.31)		0.01 (-0.22, 0.24)	
PM_2.5_					1.21*** (0.72, 1.69)		-0.03 (-0.17, 0.12)			-0.28* (-0.55, -0.00)			-0.52** (-0.91, -0.13)				0.38* (0.05, 0.71)		-0.01 (-0.39, 0.38)			-0.00 (-0.01, 0.01)			-0.05*** (-0.08, -0.02)		-0.05 (-0.21, 0.11)			0.34*** (0.21, 0.47)		-0.01 (-0.27, 0.25)			0.27*** (0.14, 0.40)		-0.01 (-0.33, 0.31)		-0.01 (-0.24, 0.22)	
SO_2_					1.24*** (0.73, 1.75)		-0.05 (-0.20, 0.11)			-0.32* (-0.62, -0.03)			-0.84* (-1.51, -0.17)				0.38* (0.03, 0.72)		-0.35 (-1.10, 0.40)			-0.00 (-0.01, 0.01)			-0.05*** (-0.08, -0.02)		-0.04 (-0.20, 0.12)			0.34*** (0.21, 0.47)		-0.04 (-0.30, 0.22)			0.27*** (0.14, 0.40)		-0.01 (-0.32, 0.31)		-0.01 (-0.24, 0.23)	
Temperature					1.21*** (0.78, 1.64)		0.03 (-0.03, 0.08)			-0.38*** (-0.55, -0.21)			-0.63*** (-0.88, -0.37)				0.35 (-0.58, 1.28)		0.47 (-0.15, 1.10)			-0.00 (-0.01, 0.01)			-0.05*** (-0.08, -0.02)		-0.04 (-0.20, 0.12)			0.34*** (0.21, 0.47)		-0.00 (-0.26, 0.26)			0.26*** (0.14, 0.39)		0.00 (-0.31, 0.32)		0.02 (-0.21, 0.25)	

C. Significant interactions: Environmental effects modified by COVID-19 period																																								
Variable	Period			LRT p		Intercept			Environment			During		Post		Env × During		Env × Post			Age		AMH			Fert. preservation			Social freezing		PGT			Protocol: Soft		Long agonist				Microflare
O_3_	365 days			0.002**		1.28*** (0.81, 1.74)			0.09 (-0.06, 0.25)			-0.47*** (-0.70, -0.24)		-0.76*** (-1.12, -0.40)		-0.33** (-0.55, -0.11)		0.10 (-0.21, 0.41)			-0.00 (-0.01, 0.01)		-0.05*** (-0.08, -0.02)			-0.04 (-0.20, 0.12)			0.34*** (0.21, 0.47)		-0.06 (-0.33, 0.21)			0.27*** (0.14, 0.40)		-0.02 (-0.35, 0.30)				-0.01 (-0.24, 0.22)
CO	365 days			0.008**		1.25*** (0.79, 1.71)			-0.06 (-0.19, 0.07)			-0.36** (-0.59, -0.14)		-0.25 (-0.58, 0.08)		0.36** (0.09, 0.62)		0.46* (0.08, 0.83)			-0.00 (-0.01, 0.01)		-0.05*** (-0.08, -0.02)			-0.04 (-0.20, 0.12)			0.34*** (0.21, 0.47)		-0.04 (-0.30, 0.22)			0.27*** (0.14, 0.40)		-0.01 (-0.33, 0.31)				-0.01 (-0.25, 0.22)
NO_2_	365 days			0.009**		1.27*** (0.73, 1.82)			-0.07 (-0.27, 0.14)			-0.41* (-0.76, -0.05)		-0.88** (-1.45, -0.32)		0.42* (0.08, 0.75)		-0.28 (-0.76, 0.21)			-0.00 (-0.01, 0.01)		-0.05*** (-0.08, -0.02)			-0.04 (-0.20, 0.12)			0.34*** (0.21, 0.47)		-0.05 (-0.31, 0.21)			0.27*** (0.14, 0.39)		-0.01 (-0.32, 0.31)				-0.01 (-0.24, 0.22)
SO_2_	365 days			0.047*		1.24*** (0.73, 1.75)			-0.05 (-0.20, 0.11)			-0.32* (-0.62, -0.03)		-0.84* (-1.51, -0.17)		0.38* (0.03, 0.72)		-0.35 (-1.10, 0.40)			-0.00 (-0.01, 0.01)		-0.05*** (-0.08, -0.02)			-0.04 (-0.20, 0.12)			0.34*** (0.21, 0.47)		-0.04 (-0.30, 0.22)			0.27*** (0.14, 0.40)		-0.01 (-0.32, 0.31)				-0.01 (-0.24, 0.23)

*p < 0.05, **p < 0.01, ***p < 0.001.

Environment: Environment, Indication, Protocol: Likelihood Ratio Test (LRT). Age and AMH: individual z-test (per year; per ng/mL). COVID: LRT (During + Post). Env × COVID: LRT (interaction terms).

Beta regression model: Mature Oocyte Rate ~ Environmental Variable × COVID Period + Age + AMH + Indication + Protocol. All environmental variables are z-score standardized. P-values for environmental, COVID, interaction, and covariate terms are from Likelihood Ratio Tests (LRT).

Regression coefficients (β) with 95% confidence intervals in parentheses. *p < 0.05, **p < 0.01, ***p < 0.001 (individual z-test).

Interpretation:

• Intercept: Log-odds when all predictors = 0 (pre-COVID, mean exposure).

• Environment: Change in log-odds per 1 SD increase during pre-COVID.

• COVID Period: Change in baseline rate during/after-COVID vs. pre-COVID.

• Interaction: Total effect in during-COVID = Environment + Env × During; Total effect in post-COVID = Environment + Env × Post.

Variables with a significant Env × COVID interaction (Likelihood Ratio Test p < 0.05). Coefficients (β) with 95% CI. *p < 0.05, **p < 0.01, ***p < 0.001 (individual z-test).

Interpretation:

• Total effect in pre-COVID = Environment β

• Total effect in during-COVID = Environment β + Env × During β

• Total effect in post-COVID = Environment β + Env × Post β

Model:

logit(μ) = β_0_ + β_1_(Env) + β_2_(During) + β_3_(Post) + β_4_(Env × During) + β_5_(Env × Post) + β_6_(Age) + β_7_(AMH) + β_8_(Indication) + β_9_(Protocol).

Exposure Periods:

• Period 1: Follicular phase (menstruation to trigger day).

• 90 days: 90-day average exposure before oocyte retrieval.

• 365 days: 365-day average exposure before oocyte retrieval.

AMH, anti-Müllerian hormone; Env, environmental variable; PM_2.5_, fine particulate matter ≤ 2.5 μm; PM10, particulate matter ≤ 10 μm; SD, standard deviation; SE, standard error; φ, precision parameter; CO, carbon monoxide; COVID-19, coronavirus disease 2019; NO_2_, nitrogen dioxide; O_3_, ozone; SO_2_, sulfur dioxide.

**Table 4:** Beta regression results: Fertilization rate with COVID-19 interaction.

A. Beta regression: p-values for model selection (fertilization rate)																	
Variable		Period		N	p-value							Indication		Protocol	Fert. method		TMSC
					Environment	Age		AMH		COVID	Env × COVID						
Period 1																	
CO		Period 1		572	0.543	0.529		0.308		0.004**	0.674	0.383		0.001**	< 0.001***		0.629
Humidity		Period 1		572	0.352	0.515		0.330		0.005**	0.845	0.401		0.001**	< 0.001***		0.600
NO_2_		Period 1		572	0.833	0.507		0.305		0.007**	0.474	0.391		0.002**	< 0.001***		0.575
O_3_		Period 1		572	0.457	0.509		0.285		0.007**	0.984	0.379		0.001**	< 0.001***		0.592
PM_10_		Period 1		572	0.563	0.513		0.311		0.003**	0.31	0.414		0.003**	< 0.001***		0.545
PM_2_._5_		Period 1		572	0.740	0.509		0.294		0.002**	0.656	0.392		0.002**	< 0.001***		0.553
SO_2_		Period 1		572	0.442	0.492		0.286		0.003**	0.584	0.405		0.003**	< 0.001***		0.434
Temperature		Period 1		572	0.524	0.516		0.301		0.003**	0.933	0.39		0.001**	< 0.001***		0.594
90 days																	
CO		90 days		1,234	0.519	0.456		0.004**		0.054	0.547	0.627		0.170	< 0.001***		0.034*
Humidity		90 days		1,234	0.912	0.468		0.004**		0.045*	0.921	0.596		0.182	< 0.001***		0.033*
NO_2_		90 days		1,234	0.752	0.465		0.004**		0.082	0.635	0.614		0.170	< 0.001***		0.036*
O_2_		90 days		1,234	0.399	0.456		0.003**		0.077	0.815	0.637		0.179	< 0.001***		0.029*
PM_10_		90 days		1,234	0.841	0.468		0.004**		0.037*	0.721	0.575		0.172	< 0.001***		0.035*
PM_2_._5_		90 days		1,234	0.836	0.464		0.004**		0.046*	0.732	0.595		0.172	< 0.001***		0.034*
SO_2_		90 days		1,234	0.763	0.453		0.004**		0.113	0.89	0.597		0.178	< 0.001***		0.037*
Temperature		90 days		1,234	0.622	0.463		0.004**		0.040*	0.613	0.625		0.167	< 0.001***		0.036*
365 days																	
CO		365 days		1,234	0.012*	0.385		0.003**		0.110	0.803	0.575		0.186	< 0.001***		0.033*
Humidity		365 days		1,234	0.413	0.433		0.004**		0.228	0.522	0.598		0.188	< 0.001***		0.030*
NO_2_		365 days		1,234	0.224	0.424		0.003**		0.388	0.718	0.619		0.171	< 0.001***		0.036*
O_3_		365 days		1,234	0.226	0.423		0.003**		0.577	0.647	0.617		0.165	< 0.001***		0.039*
PM_10_		365 days		1,234	0.724	0.466		0.003**		0.075	0.645	0.593		0.179	< 0.001***		0.038*
PM_2_._5_		365 days		1,234	0.167	0.42		0.003**		0.174	0.808	0.614		0.169	< 0.001***		0.036*
SO_2_		365 days		1,234	0.309	0.414		0.003**		0.219	0.765	0.628		0.175	< 0.001***		0.030*
Temperature		365 days		1,234	0.359	0.449		0.003**		0.078	0.483	0.604		0.185	< 0.001***		0.038*

B. Beta regression coefficients: All variables (fertilization rate)																	
Variable	Period	Intercept	Environment	COVID Period		Interaction		Age	AMH	Fert. preservation	PGT	Protocol: Soft	Long agonist	Microflare	Fert: all IVF	Fert: IVF+ICSI	TMSC
				During	Post	Env × During	Env × Post										
CO	Period 1	1.31** (0.47, 2.16)	0.05 (-0.12, 0.22)	0.08 (-0.17, 0.34)	0.43** (0.17, 0.70)	-0.08 (-0.32, 0.16)	-0.12 (-0.31, 0.08)	0.01 (-0.01, 0.03)	-0.03 (-0.09, 0.03)	-0.22 (-0.52, 0.08)	0.66** (0.18, 1.13)	0.48*** (0.28, 0.69)	0.24 (-0.11, 0.60)	0.20 (-0.06, 0.47)	-1.11*** (-1.61, -0.61)	-0.59*** (-0.79, -0.39)	0.02 (-0.04, 0.08)
Humidity	Period 1	1.41*** (0.58, 2.24)	0.10 (-0.14, 0.35)	-0.02 (-0.29, 0.25)	0.34* (0.05, 0.63)	-0.06 (-0.33, 0.21)	-0.09 (-0.37, 0.20)	0.01 (-0.01, 0.03)	-0.03 (-0.09, 0.03)	-0.22 (-0.52, 0.08)	0.63** (0.20, 1.06)	0.49*** (0.28, 0.70)	0.22 (-0.14, 0.58)	0.21 (-0.06, 0.47)	-1.12*** (-1.62, -0.62)	-0.60*** (-0.79, -0.40)	0.02 (-0.04, 0.08)
NO_2_	Period 1	1.23** (0.35, 2.11)	0.11 (-0.11, 0.33)	0.15 (-0.15, 0.46)	0.47** (0.14, 0.81)	-0.15 (-0.42, 0.12)	-0.18 (-0.46, 0.11)	0.01 (-0.01, 0.03)	-0.03 (-0.09, 0.03)	-0.22 (-0.52, 0.08)	0.65** (0.18, 1.12)	0.47*** (0.26, 0.68)	0.21 (-0.14, 0.57)	0.19 (-0.07, 0.46)	-1.11*** (-1.61, -0.62)	-0.59*** (-0.79, -0.39)	0.02 (-0.04, 0.08)
O_3_	Period 1	1.34** (0.43, 2.25)	0.01 (-0.35, 0.38)	0.04 (-0.34, 0.42)	0.38 (-0.02, 0.77)	0.02 (-0.36, 0.41)	0.03 (-0.36, 0.42)	0.01 (-0.01, 0.03)	-0.03 (-0.09, 0.03)	-0.22 (-0.52, 0.08)	0.68** (0.18, 1.19)	0.49*** (0.28, 0.69)	0.23 (-0.12, 0.58)	0.20 (-0.06, 0.47)	-1.12*** (-1.59, -0.64)	-0.59*** (-0.79, -0.39)	0.02 (-0.04, 0.08)
PM_10_	Period 1	1.27** (0.46, 2.08)	0.11 (-0.12, 0.33)	0.07 (-0.15, 0.30)	0.43*** (0.20, 0.67)	-0.19 (-0.44, 0.06)	-0.12 (-0.37, 0.13)	0.01 (-0.01, 0.03)	-0.03 (-0.09, 0.03)	-0.22 (-0.52, 0.08)	0.62** (0.23, 1.01)	0.46*** (0.26, 0.67)	0.21 (-0.15, 0.56)	0.20 (-0.06, 0.47)	-1.13*** (-1.62, -0.63)	-0.60*** (-0.80, -0.40)	0.02 (-0.04, 0.08)
PM_2_._5_	Period 1	1.28** (0.45, 2.11)	0.08 (-0.09, 0.24)	0.10 (-0.14, 0.34)	0.45*** (0.20, 0.70)	-0.08 (-0.30, 0.14)	-0.11 (-0.34, 0.12)	0.01 (-0.01, 0.03)	-0.03 (-0.09, 0.03)	-0.21 (-0.52, 0.09)	0.67** (0.20, 1.15)	0.47*** (0.26, 0.68)	0.23 (-0.13, 0.58)	0.20 (-0.06, 0.47)	-1.12*** (-1.62, -0.63)	-0.60*** (-0.80, -0.40)	0.02 (-0.04, 0.08)
SO_2_	Period 1	1.14** (0.28, 2.00)	0.12 (-0.07, 0.30)	0.19 (-0.11, 0.49)	0.57** (0.18, 0.95)	-0.15 (-0.45, 0.14)	-0.09 (-0.47, 0.29)	0.01 (-0.01, 0.03)	-0.03 (-0.09, 0.03)	-0.21 (-0.51, 0.09)	0.67** (0.20, 1.14)	0.46*** (0.25, 0.67)	0.22 (-0.14, 0.57)	0.21 (-0.06, 0.48)	-1.15*** (-1.64, -0.65)	-0.61*** (-0.80, -0.41)	0.03 (-0.03, 0.09)
Temperature	Period 1	1.37** (0.52, 2.22)	0.06 (-0.14, 0.27)	0.02 (-0.23, 0.27)	0.38** (0.12, 0.64)	-0.05 (-0.29, 0.20)	-0.04 (-0.28, 0.21)	0.01 (-0.01, 0.03)	-0.03 (-0.09, 0.03)	-0.22 (-0.52, 0.08)	0.66** (0.18, 1.14)	0.49*** (0.28, 0.70)	0.23 (-0.12, 0.58)	0.21 (-0.06, 0.48)	-1.13*** (-1.62, -0.65)	-0.59*** (-0.79, -0.39)	0.02 (-0.04, 0.08)
CO	90 days	1.27*** (0.68, 1.86)	-0.07 (-0.22, 0.09)	-0.00 (-0.24, 0.23)	0.16 (-0.09, 0.40)	0.09 (-0.09, 0.27)	0.01 (-0.18, 0.19)	-0.01 (-0.02, 0.01)	-0.04** (-0.07, -0.01)	-0.14 (-0.32, 0.04)	-0.06 (-0.34, 0.22)	0.18* (0.02, 0.33)	0.17 (-0.11, 0.46)	0.17 (-0.02, 0.36)	-0.75*** (-1.07, -0.43)	-0.37*** (-0.50, -0.24)	0.06** (0.02, 0.10)
Humidity	90 days	1.25*** (0.65, 1.84)	0.04 (-0.19, 0.26)	0.01 (-0.28, 0.30)	0.19 (-0.11, 0.49)	-0.04 (-0.28, 0.20)	-0.06 (-0.31, 0.20)	-0.01 (-0.02, 0.01)	-0.04** (-0.07, -0.01)	-0.15 (-0.33, 0.03)	-0.06 (-0.34, 0.22)	0.17* (0.01, 0.33)	0.16 (-0.13, 0.44)	0.17 (-0.02, 0.36)	-0.75*** (-1.07, -0.42)	-0.36*** (-0.49, -0.23)	0.06** (0.02, 0.10)
NO_2_	90 days	1.31*** (0.71, 1.91)	-0.09 (-0.28, 0.11)	-0.04 (-0.33, 0.25)	0.11 (-0.19, 0.42)	0.11 (-0.11, 0.33)	0.06 (-0.18, 0.29)	-0.01 (-0.02, 0.01)	-0.04** (-0.07, -0.01)	-0.15 (-0.33, 0.04)	-0.06 (-0.35, 0.22)	0.18* (0.02, 0.33)	0.16 (-0.12, 0.45)	0.17 (-0.02, 0.36)	-0.75*** (-1.07, -0.42)	-0.37*** (-0.50, -0.23)	0.06** (0.02, 0.10)
O_3_	90 days	1.23*** (0.63, 1.83)	0.03 (-0.14, 0.20)	0.02 (-0.22, 0.26)	0.18 (-0.07, 0.43)	-0.02 (-0.21, 0.17)	0.03 (-0.16, 0.22)	-0.01 (-0.02, 0.01)	-0.04** (-0.07, -0.01)	-0.14 (-0.32, 0.04)	-0.06 (-0.34, 0.22)	0.17* (0.01, 0.33)	0.17 (-0.12, 0.45)	0.17 (-0.02, 0.36)	-0.75*** (-1.08, -0.43)	-0.37*** (-0.50, -0.23)	0.06** (0.02, 0.10)
PM_10_	90 days	1.24*** (0.67, 1.81)	-0.06 (-0.22, 0.11)	0.02 (-0.18, 0.23)	0.20 (-0.01, 0.42)	0.08 (-0.11, 0.27)	0.08 (-0.11, 0.27)	-0.01 (-0.02, 0.01)	-0.04** (-0.07, -0.01)	-0.16 (-0.34, 0.02)	-0.05 (-0.33, 0.23)	0.18* (0.02, 0.34)	0.15 (-0.13, 0.44)	0.17 (-0.02, 0.36)	-0.74*** (-1.07, -0.42)	-0.36*** (-0.49, -0.23)	0.06** (0.02, 0.10)
PM_2_._5_	90 days	1.26*** (0.68, 1.83)	-0.06 (-0.21, 0.09)	0.01 (-0.21, 0.23)	0.18 (-0.05, 0.42)	0.07 (-0.11, 0.26)	0.07 (-0.13, 0.26)	-0.01 (-0.02, 0.01)	-0.04** (-0.07, -0.01)	-0.15 (-0.33, 0.03)	-0.06 (-0.34, 0.22)	0.18* (0.02, 0.33)	0.15 (-0.13, 0.44)	0.17 (-0.02, 0.36)	-0.74*** (-1.07, -0.41)	-0.36*** (-0.49, -0.23)	0.06** (0.02, 0.10)
SO_2_	90 days	1.29*** (0.68, 1.89)	-0.05 (-0.23, 0.13)	-0.02 (-0.32, 0.28)	0.18 (-0.18, 0.53)	0.05 (-0.18, 0.29)	0.08 (-0.27, 0.43)	-0.01 (-0.02, 0.01)	-0.04** (-0.07, -0.01)	-0.15 (-0.33, 0.03)	-0.06 (-0.34, 0.23)	0.17* (0.02, 0.33)	0.15 (-0.14, 0.44)	0.17 (-0.02, 0.36)	-0.74*** (-1.07, -0.42)	-0.36*** (-0.49, -0.23)	0.06** (0.02, 0.10)
Temperature	90 days	1.23*** (0.66, 1.81)	0.07 (-0.08, 0.23)	0.03 (-0.17, 0.22)	0.20 (-0.01, 0.41)	-0.09 (-0.27, 0.08)	-0.04 (-0.22, 0.14)	-0.01 (-0.02, 0.01)	-0.04** (-0.07, -0.01)	-0.14 (-0.32, 0.04)	-0.06 (-0.34, 0.22)	0.18* (0.02, 0.33)	0.17 (-0.12, 0.45)	0.17 (-0.02, 0.37)	-0.75*** (-1.07, -0.43)	-0.37*** (-0.50, -0.24)	0.06** (0.02, 0.10)
CO	365 days	1.60*** (0.95, 2.25)	-0.22 (-0.46, 0.02)	-0.34 (-0.74, 0.07)	-0.38 (-0.91, 0.15)	0.08 (-0.23, 0.38)	-0.10 (-0.58, 0.38)	-0.01 (-0.02, 0.01)	-0.04** (-0.07, -0.01)	-0.16 (-0.34, 0.02)	-0.02 (-0.30, 0.26)	0.17* (0.01, 0.33)	0.13 (-0.16, 0.43)	0.18 (-0.02, 0.37)	-0.67*** (-1.00, -0.34)	-0.37*** (-0.50, -0.24)	0.06** (0.02, 0.10)
Humidity	365 days	1.54*** (0.75, 2.34)	0.19 (-0.19, 0.58)	-0.28 (-0.92, 0.35)	-0.00 (-0.66, 0.66)	-0.10 (-0.52, 0.31)	-0.31 (-0.79, 0.17)	-0.01 (-0.02, 0.01)	-0.04** (-0.07, -0.01)	-0.15 (-0.33, 0.02)	-0.02 (-0.31, 0.26)	0.17* (0.01, 0.33)	0.14 (-0.15, 0.44)	0.17 (-0.02, 0.37)	-0.72*** (-1.05, -0.39)	-0.37*** (-0.50, -0.24)	0.06** (0.02, 0.10)
NO_2_	365 days	1.51*** (0.73, 2.28)	-0.18 (-0.57, 0.20)	-0.24 (-0.83, 0.35)	0.01 (-0.67, 0.69)	0.04 (-0.42, 0.49)	0.25 (-0.30, 0.80)	-0.01 (-0.02, 0.01)	-0.04** (-0.07, -0.01)	-0.15 (-0.33, 0.03)	-0.03 (-0.32, 0.25)	0.17* (0.01, 0.33)	0.15 (-0.14, 0.44)	0.18 (-0.01, 0.38)	-0.70*** (-1.04, -0.37)	-0.36*** (-0.49, -0.23)	0.06** (0.02, 0.10)
O_3_	365 days	1.43*** (0.81, 2.04)	0.15 (-0.09, 0.40)	-0.14 (-0.48, 0.21)	0.05 (-0.35, 0.46)	-0.09 (-0.37, 0.20)	-0.18 (-0.51, 0.16)	-0.01 (-0.02, 0.01)	-0.04** (-0.07, -0.01)	-0.15 (-0.33, 0.03)	-0.04 (-0.32, 0.24)	0.17* (0.02, 0.33)	0.15 (-0.13, 0.44)	0.18 (-0.02, 0.38)	-0.70*** (-1.03, -0.37)	-0.36*** (-0.49, -0.23)	0.06** (0.02, 0.09)
PM_10_	365 days	1.36*** (0.73, 1.99)	-0.11 (-0.37, 0.15)	-0.08 (-0.44, 0.28)	0.09 (-0.28, 0.45)	0.14 (-0.19, 0.47)	0.11 (-0.19, 0.41)	-0.01 (-0.02, 0.01)	-0.04** (-0.07, -0.01)	-0.15 (-0.33, 0.03)	-0.06 (-0.34, 0.22)	0.18* (0.02, 0.33)	0.15 (-0.14, 0.44)	0.17 (-0.03, 0.36)	-0.73*** (-1.06, -0.39)	-0.37*** (-0.50, -0.23)	0.06** (0.02, 0.10)
PM_2_._5_	365 days	1.52*** (0.78, 2.27)	-0.20 (-0.55, 0.16)	-0.27 (-0.82, 0.27)	-0.10 (-0.68, 0.48)	0.09 (-0.35, 0.53)	0.15 (-0.30, 0.61)	-0.01 (-0.02, 0.01)	-0.04** (-0.07, -0.01)	-0.15 (-0.33, 0.03)	-0.05 (-0.33, 0.23)	0.17* (0.02, 0.33)	0.15 (-0.14, 0.44)	0.18 (-0.02, 0.38)	-0.70*** (-1.04, -0.37)	-0.36*** (-0.49, -0.23)	0.06** (0.02, 0.10)
SO_2_	365 days	1.32*** (0.66, 1.98)	-0.06 (-0.32, 0.20)	-0.09 (-0.51, 0.33)	0.17 (-0.44, 0.78)	-0.14 (-0.52, 0.24)	0.10 (-0.56, 0.77)	-0.01 (-0.02, 0.01)	-0.04** (-0.07, -0.01)	-0.15 (-0.33, 0.03)	-0.03 (-0.31, 0.25)	0.17* (0.01, 0.33)	0.16 (-0.13, 0.45)	0.19 (-0.01, 0.38)	-0.72*** (-1.05, -0.39)	-0.37*** (-0.50, -0.23)	0.06** (0.02, 0.10)
Temperature	365 days	1.25*** (0.69, 1.82)	0.05 (-0.05, 0.16)	0.08 (-0.13, 0.29)	0.23 (-0.03, 0.50)	-0.62 (-1.45, 0.21)	-0.17 (-0.76, 0.41)	-0.01 (-0.02, 0.01)	-0.04** (-0.07, -0.01)	-0.15 (-0.33, 0.03)	-0.05 (-0.33, 0.24)	0.17* (0.01, 0.33)	0.14 (-0.15, 0.43)	0.17 (-0.03, 0.36)	-0.73*** (-1.05, -0.40)	-0.37*** (-0.50, -0.24)	0.06** (0.02, 0.09)
C. Significant interactions: Environmental effects modified by COVID-19 period (fertilization rate)																	

Beta regression model: Fertilization Rate ~ Environmental Variable × COVID Period + Age + AMH + Indication + Protocol + Fertilization method + TMSC. All environmental variables are z-score standardized. P-values for environmental, COVID, interaction, and covariate terms are from Likelihood Ratio Tests (LRT).

*p < 0.05, **p < 0.01, ***p < 0.001

Environment: Environment, Indication, Protocol, Fert method, TMSC: Likelihood Ratio Test (LRT). Age and AMH: individual z-test (per year; per ng/mL). COVID: LRT (During + Post). Env × COVID: LRT (interaction terms).

Regression coefficients (β) with 95% confidence intervals in parentheses. *p < 0.05, **p < 0.01, ***p < 0.001 (individual z-test). TMSC was rescaled to per 100 million motile sperm (effect per 100 million units).

Interpretation:

• Intercept: Log-odds when all predictors = 0 (pre-COVID, mean exposure).

• Environment: Change in log-odds per 1 SD increase during pre-COVID.

• COVID Period: Change in baseline rate during/after-COVID vs. pre-COVID.

• Interaction: Total effect during-COVID = Environment + Env × During; Total effect post-COVID = Environment + Env × Post

• φ: Precision parameter.

No significant Env × COVID interactions were detected (all Wald test p ≥ 0.05).

Model Specification

Model: logit(μ) = β_0_ + β_1_(Env) + β_2_(During) + β_3_(Post) + β_4_(Age) + β_5_(AMH) + β_6_(Env × During) + β_7_(Env × Post).

Exposure Periods:

• Period 1: Follicular phase (menstruation to trigger day).

• 90 days: 90-day average exposure before oocyte retrieval.

• 365 days: 365-day average exposure before oocyte retrieval.

AMH, anti-Müllerian hormone; Env, environmental variable; PM_2.5_, fine particulate matter ≤ 2.5 μm; PM10, particulate matter ≤ 10 μm; SD, standard deviation; SE, standard error; φ, precision parameter; CO, carbon monoxide; COVID-19, coronavirus disease 2019; NO_2_, nitrogen dioxide; O_3_, ozone; SO_2_, sulfur dioxide.

### 2.2 Ethical approval

The study protocol was approved by the Institutional Review Board of Yonsei University College of Medicine, Seoul, South Korea (approval number: #4-2025-0602).

### 2.3 Oocyte retrieval procedure and laboratory procedures

COS was initiated using individualized gonadotropin regimens, including recombinant follicle-stimulating hormone (Follitrope^®^, LG Chem; Puregon^®^, MSD; or Rekovelle^®^, Ferring) or human menopausal gonadotropin (IVF-M HP^®^, LG Chem). Follicular growth was monitored using serial transvaginal or transrectal ultrasonography and serum estradiol measurements. Final oocyte maturation was triggered when at least two leading follicles reached ≥18 mm in diameter, using either recombinant human chorionic gonadotropin (Ovidrel^®^, Merck Serono) or a gonadotropin-releasing hormone (GnRH) agonist (Decapeptyl^®^, Ferring; or Leuplin^®^, Takeda), depending on the stimulation protocol and risk of ovarian hyperstimulation syndrome. A GnRH antagonist protocol (Cetrotide^®^, Merck Serono; or Ganilever^®^, LG Chem) was used in most cases. Oocyte retrieval was performed 34 h after the trigger injection under transvaginal ultrasound and light intravenous sedation. Mature oocytes were defined as MII, and fertilized oocytes were defined as the presence of two pronuclei at 16–18 h post-insemination. Embryo morphology was evaluated daily using standard morphological criteria, and cleavage- or blastocyst-stage embryos were either cryopreserved or transferred according to patient preference and clinical guidance. All laboratory protocols were performed by two senior embryologists using identical systems throughout the study period.

### 2.4 Classification of COVID-19 period

Each OPU cycle was categorized into one of the three distinct COVID-19 periods according to the OPU date and major public health policy milestones in South Korea. Cycles with OPU dates before March 1, 2020 were classified as pre-COVID, representing the period prior to the first confirmed domestic outbreak and before implementation of nationwide social distancing measures. Cycles performed between March 1, 2020 and December 31, 2022 were designated as during-COVID, corresponding to the active pandemic response phase, characterized by restricted mobility and healthcare access. Cycles conducted after January 1, 2023 were classified as post-COVID, reflecting the resumption of normal clinical and societal conditions following the official easing of pandemic-related restrictions. This classification reflects major shifts in population behavior and environmental exposure patterns, which may have influenced reproductive health outcomes.

### 2.5 Environmental exposure assessment

Environmental data were obtained from national air quality monitoring stations in Seodaemun-gu, Seoul, South Korea. Eight environmental factors were evaluated: particulate matter (PM_10_, PM_2.5_), gaseous pollutants (NO_2_, O_3_, CO, SO_2_), temperature, and humidity. For gaseous pollutants (NO_2_, O_3_, CO, and SO_2_), concentrations were converted from parts per million (ppm) to parts per billion (ppb) by multiplying with 1000. All environmental variables were standardized using z-score transformation based on the pre-COVID period distribution so that effect sizes could be interpreted relative to pre-pandemic norms and compared directly across pollutants measured on different scales. Environmental exposure was evaluated across multiple biologically relevant time windows and calculated individually for each participant based on the actual OPU date.

For mature oocyte and fertilization rate analyses, three exposure windows were defined. Period 1 (follicular phase) was calculated from the onset of menstruation to oocyte retrieval, with a duration of approximately 12–14 days per patient), capturing the environmental influences during follicular development and oocyte maturation. The 90-day lag represented the mean environmental exposure 90 days before OPU, reflecting the environmental impacts during late oogenesis. The 365-day lag represented the mean environmental exposure in the 365 days before OPU, reflecting longer-term environmental impacts on ovarian reserve.

For the pregnancy outcome analysis, three additional exposure windows were included, resulting in a total of six windows. Period 2 (fertilization window) was defined from the OPU date to embryo transfer (ET), encompassing exposure during fertilization and preimplantation embryo development (3–5 days depending on the day of transfer). Period 3 (implantation window) was measured from ET to OPU + 14 days, representing exposure during implantation and early pregnancy. Finally, Period 4 (full IVF cycle) was defined as the period from the start date to OPU + 14 days, reflecting cumulative exposure across the entire IVF treatment period.

### 2.6 Statistical analysis

We evaluated the associations among IVF outcomes (mature oocyte rate, fertilization rate, and clinical pregnancy) and eight environmental exposures, including particulate matter (PM_10_, PM_2.5_), gaseous pollutants (NO_2_, O_3_, CO, SO_2_), temperature, and humidity. Exposure levels were summarized across multiple biologically relevant windows to reflect temporal variations in environmental effects on reproductive processes. For the analyses of mature oocyte and fertilization rates, we considered three exposure windows: (1) the follicular phase (approximately 12–14 days before oocyte retrieval), capturing environmental influences during follicular development; (2) a 90-day lag period, reflecting environmental impacts during late oogenesis; and (3) a 365-day lag period, representing longer-term environmental impacts on ovarian reserve.

For the pregnancy outcome analysis, six exposure windows were evaluated: (1) the follicular phase; (2) the fertilization window (3–5 days following oocyte retrieval), capturing exposures during fertilization and early embryonic development; (3) the implantation window (9–11 days following embryo transfer), capturing exposures during implantation and early pregnancy establishment; (4) the full IVF cycle (approximately 26–28 days), capturing cumulative effects throughout the entire treatment period; (5) a 90-day lag period; and (6) a 365-day lag period. Environmental exposures were calculated as mean values over each exposure window for individual patients. Continuous variables are summarized as means with standard deviations and categorical variables as frequencies and percentages. Differences in continuous variables across the COVID-19 periods were assessed using the Kruskal–Wallis test, whereas differences in the categorical variables were evaluated using the chi-square test. Pairwise comparisons across the three COVID-19 periods were adjusted for multiple testing using the Bonferroni method. Multivariable beta regression models for mature oocyte rate and fertilization rate were additionally adjusted for maternal age, anti-Müllerian hormone (AMH), treatment indication, and controlled ovarian stimulation protocol. For the fertilization rate and clinical pregnancy analyses, fertilization method and total motile sperm count were further included as covariates. The clinical pregnancy model was adjusted for maternal age, treatment indication, stimulation protocol, fertilization method, and total motile sperm count; AMH was not included because of missing data. Because multiple oocyte pickup cycles from the same patient were included, cluster-robust (sandwich) standard errors were applied using the patient identifier as the clustering variable. Multicollinearity was assessed using variance inflation factors.

To minimize potential confounding, models were adjusted for maternal age, AMH, treatment indications (infertility, cancer-related fertility preservation, elective oocyte cryopreservation, and PGT-related), ovarian stimulation protocols (GnRH antagonist, soft, long, and microflare), fertilization methods (all IVF, all ICSI, and IVF+ICSI), and total motile sperm count (TMSC) as a continuous variable. Calendar year was considered as a covariate for temporal trends but was not included in the final models because it was highly collinear with the COVID-19 period variable.

Beta regression models were used to assess the associations of COVID-19 periods with mature oocyte and fertilization rates. For the analysis, the observations with boundary values (0 or 1) were transformed using the adjustment {y(n−1)+0.5}/n, where *y* is the observed proportion, and *n* is the sample size, following the approach of Smithson and Verkuilen (2006). Associations between COVID-19 periods and the pregnancy outcome were evaluated using logistic regression models, with the binary outcome coded as intrauterine pregnancy versus biochemical pregnancy or non-pregnancy. To evaluate whether environmental exposures had differential effects across COVID-19 periods, interaction terms between each environmental variable and COVID-19 period were included in the models.

All statistical analyses were performed using R version 4.5.1 (R Foundation for Statistical Computing, Vienna, Austria). All statistical tests were two-sided with a significance level of 0.05.

## 3 Results

A total of 2162 oocyte retrieval cycles were identified during the study period. Complete-case analysis yielded 1, 871 cycles for the mature oocyte rate analysis. The full cohort of 2, 162 cycles (pre-COVID, n = 469; during-COVID, n = 951; post-COVID, n = 742) is characterized in **Table 1**. The mean maternal age differed significantly among the three groups (p < 0.001), with patients in the post-COVID group being the oldest (36.71 ± 5.81 years) compared with those in the pre-COVID (34.49 ± 6.24 years) and during-COVID (36.00 ± 5.55 years) groups. Serum AMH levels showed a modest decline over time (p = 0.012).

**Figure 1:**
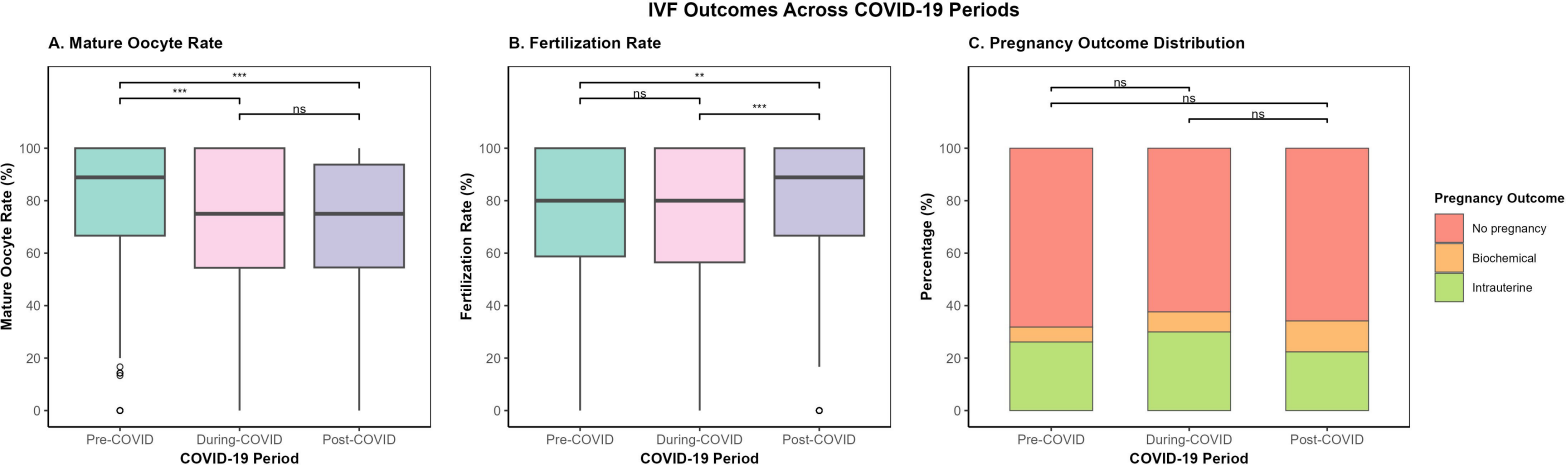
Oocyte retrieval cycle outcomes across the COVID-19 periods. **(A)** Mature oocyte rate (%), **(B)** fertilization rate (%), and **(C)** clinical pregnancy outcome distribution (%) across the pre-, during-, and post-COVID periods. Box plots represent median values and interquartile ranges; whiskers indicate the 5th–95th percentiles. Bars denote pairwise *post-hoc* comparisons (ns, not significant; **p < 0.01; ***p < 0.001). COVID-19, coronavirus disease 2019.

Regarding laboratory outcomes, the mature oocyte rate was the highest pre-COVID (79.6 ± 25.5%) and declined during- (71.7 ± 28.5%) and after- (70.1 ± 26.9%) COVID (p < 0.001). Conversely, the fertilization rate gradually increased from 74.0 ± 30.2% pre-COVID to 80.3 ± 26.2% post-COVID (p < 0.001). Pregnancy outcomes did not show significant differences across the three periods (p = 0.132) (**Figure 1**).

Wald chi-square tests revealed a significant overall effect of the COVID-19 period on mature oocyte rate (χ^²^ = 43.41, df = 2, p < 0.001) (**Table 2**). Both the during-COVID and post-COVID groups showed significantly lower mature oocyte rates than those of the pre-COVID group (pre vs. during: χ^²^ = 21.39, p < 0.001; pre vs. post: χ^²^ = 41.34, p < 0.001). In the beta regression model, the during-COVID group was associated with 27% lower odds of obtaining mature oocytes than in the pre-COVID group (β = −0.32, 95% CI −0.45 to −0.19, p < 0.001). Similarly, the post-COVID group showed a 36% reduction in odds (β = −0.45, 95% CI −0.59 to −0.31, p < 0.001). Among covariates, elevated serum AMH was also inversely associated with mature oocyte rate (β = −0.05, 95% CI −0.08 to −0.02, p < 0.001).

The fertilization rate also differed significantly across pandemic periods (Wald χ² = 8.98, df = 2, p = 0.015) (**Table 2**). Unlike the mature oocyte rate, the fertilization rate was significantly higher in the post-COVID group than in the during-COVID group (χ² = 7.62, p = 0.017); the comparison with the pre-COVID baseline (χ² = 4.74, p = 0.089) did not reach statistical significance after correction for multiple comparisons. In the beta regression model, the post-COVID group was associated with 25% higher odds of successful fertilization than in the pre-COVID group (β = 0.22, 95% CI 0.02 to 0.42, p = 0.030). The during-COVID group showed a non-significant trend toward increased odds (β = 0.05, 95% CI −0.14 to 0.24, p = 0.637).

The overall pregnancy rate (including biochemical and intrauterine pregnancies) did not demonstrate a significant difference across the three pandemic periods (Wald χ^²^ = 2.12, df = 2, p = 0.346) (**Table 2**). Neither the during-COVID nor post-COVID groups showed a significant difference in pregnancy odds compared to the pre-COVID baseline. However, maternal age was inversely associated with pregnancy rate (β = −0.15, 95% CI −0.20 to −0.10, p < 0.001), with each additional year associated with a 14% reduction in the odds of achieving pregnancy.

**Figure 2:**
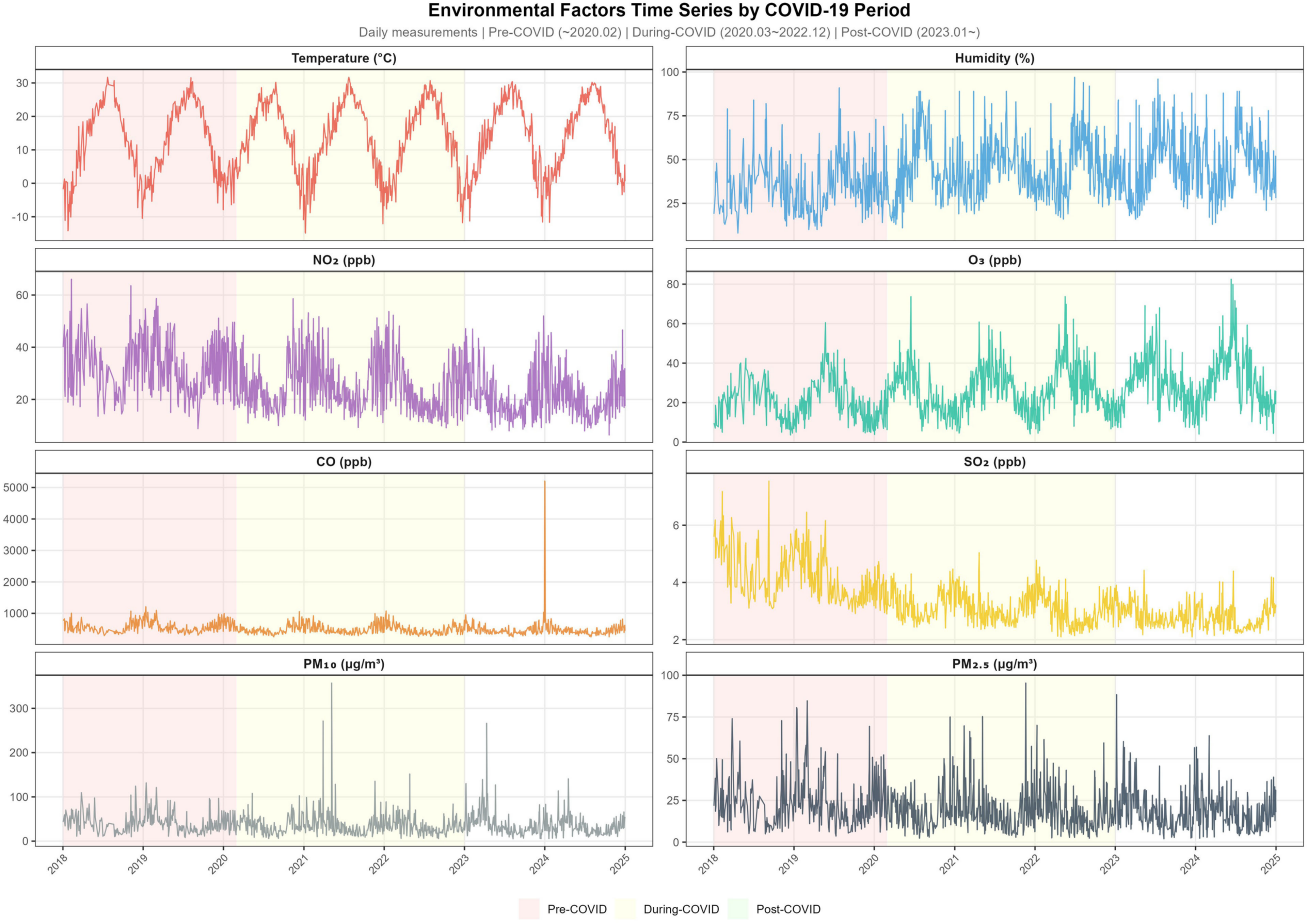
Time series of environmental factor by COVID-19 period. Daily measurements of ambient temperature (°C), relative humidity (%), nitrogen dioxide (NO_2_, ppb), ozone (O_3_, ppb), carbon monoxide (CO, ppb), sulfur dioxide (SO_2_, ppb), particulate matter ≤ 10 μm (PM_10_, μg/m³), and particulate matter ≤ 2.5 μm (PM_2.5_, μg/m³) in Seoul, Republic of Korea, from January 2018 to December 2024. Shaded backgrounds indicate the three COVID-19 periods: Pre-COVID (before March 1, 2020), During-COVID (March 1, 2020 to December 31, 2022), and Post-COVID (after January 1, 2023). The figure illustrates pronounced seasonal variability in temperature and humidity, a general decline in primary air pollutants (NO_2_, CO, SO_2_, PM_10_, PM_2.5_) during and after the pandemic period, and a relative increase in O_3_ concentrations across the study period. COVID-19, coronavirus disease 2019; ppb, parts per billion.

Across all three COVID-19 periods, significant differences were observed in the ovulation period, 90-day, and 365-day average concentrations of all measured environmental variables, including temperature, humidity, PM_10_, PM_2.5_, NO_2_, O_3_, CO, and SO_2_ (all p < 0.001) (**Figure 2**). Primary air pollutants (PM_10_, PM_2.5_, NO_2_, CO, and SO_2_) demonstrated the highest concentrations in the pre-COVID group, followed by a marked decline in the during- and after-COVID groups. In contrast, O_3_ concentrations, mean temperature, and mean humidity displayed an increasing trend from pre-COVID to post-COVID. These distinct shifts in ambient exposure profiles across the pandemic phases provide a relevant contextual basis for examining the potential interaction effects between environmental exposures and COVID-19 on reproductive outcomes.

**Table 3** summarizes the p-values for environmental variables and their interactions with the COVID-19 periods in the beta regression models for the mature oocyte rate (n = 1871). Across Period 1 and the 90-day window, no environmental factors showed significant interactions with COVID-19. During these short windows, the COVID-19 period was strongly associated with lower mature oocyte rates, whereas the main environmental effects were typically null. In contrast, significant interactions between environmental factors and COVID-19 period were observed for long-term (365-day) exposures on the Likelihood Ratio Test (LRT), including O_3_ (LRT p = 0.002), CO (LRT p = 0.008), NO_2_ (LRT p = 0.009), and SO_2_ (LRT p = 0.047), indicating that the impact of these pollutants on mature oocyte rate differed by COVID-19 period. For O_3_, the main association was weakly positive in the pre-COVID reference (β = 0.09), shifted sharply negative during COVID-19 owing to a significant negative interaction (β ≈ 0.09 − 0.33), and reverted to a weak positive association in the post-COVID group (β ≈ 0.09 + 0.10). For CO, a negative pre-COVID main effect (β = −0.06) was offset by a significant positive interaction during COVID-19 (β ≈ −0.06 + 0.36) and further strengthened after the pandemic (β ≈ −0.06 + 0.46). For NO_2_, the weakly negative pre-COVID main effect (β = −0.07) was reversed by a significant positive interaction during COVID-19 (β ≈ −0.07 + 0.42) and returned toward a modestly negative association in the post-COVID group (β ≈ −0.07 − 0.28). These findings suggest that chronic exposure to specific air pollutants influences oocyte maturation and alters the direction and magnitude of COVID period-specific effects, implying heightened susceptibility of the ovarian microenvironment to long-term air quality conditions.

Regarding the fertilization rate, no environmental factors showed a significant interaction with the COVID-19 period across all exposure windows (ovulation period, 90-day, and 365-day) (**Table 4**). This observation suggests that the improvements in fertilization rates in the post-COVID group were not attributable to changes in ambient pollution levels.

For clinical pregnancy outcomes, five environment–COVID-19 period interactions reached nominal statistical significance on the Likelihood Ratio Test (LRT) across the evaluated windows (Table 5): SO_2_ in the 90-day exposure window (p = 0.014), with a stronger positive association in the post-COVID group; temperature in the 365-day exposure window (p = 0.013); O_3_ in the 365-day exposure window (p = 0.020); humidity in Period 3 (p = 0.040); and NO_2_ in the 90-day exposure window (p = 0.049). Among these, the SO_2_ 90-day interaction showed the strongest statistical evidence. The remaining interactions should be interpreted with caution given the multiple exposure windows and environmental variables evaluated.

## 4 Discussion

In this retrospective cohort study, we observed that the COVID-19 period was markedly associated with IVF outcomes, with the mature oocyte rate declining and the fertilization rate increasing after the pandemic. In addition, we found novel evidence of significant interaction effects between long-term exposure to ambient environmental factors and the societal context of the pandemic on IVF outcomes. Critically, chronic pollutant exposure to O_3_, CO, NO_2_, and COVID-19 demonstrated interaction effects on the mature oocyte rate.

The progressive decline in the mature oocyte rate from pre- (79.5%) to post-COVID (69.8%) persisted after adjusting for maternal age and AMH levels, suggesting independent period effects. Treatment delays and postponed cycles during pandemic restrictions might have resulted in patient populations with longer infertility duration and potentially more compromised ovarian function by the time treatment was pursued (28, 29). A significant increase in maternal age and a decline in AMH level across periods reflects demographic trends and treatment delays. Pandemic-related psychological stress, lifestyle changes, diet alterations, and sedentary behavior can affect the hypothalamic-pituitary-ovarian axis and potentially impacting folliculogenesis and oocyte development through neuroendocrine pathways (30, 31).

In contrast, the fertilization rate improved over time, with significantly higher odds of successful fertilization in the post-COVID group than in the pre-COVID group, while clinical pregnancy rates remained stable. This trend can be explained by several mechanisms. First, sperm quality may have improved during the pandemic owing to reductions in environmental and lifestyle stressors (32). Second, oocyte selection bias is plausible, as the overall oocyte quality declined during the pandemic period and only robust oocytes were successfully inseminated and matured, leading to a higher proportion of fertilization among the smaller selective MII group. Finally, continued optimization of laboratory workflows and gamete-handling protocols throughout the study period may have increased fertilization efficiency. The lack of significant environmental interactions for fertilization rate suggests that this outcome may be less susceptible to ambient air pollution effects, possibly because of the more controlled laboratory environment in which fertilization occurs during this critical window (33, 34).

The documented environmental changes during the pandemic period in Seoul align with global trends in that primary pollutants derived from vehicular and industrial emissions (PM, NO_2_, CO, and SO_2_) decreased substantially, whereas O_3_, temperature, and humidity increased (21, 35–37). These contextual changes motivated our interaction analyses to test whether the effects of air pollution on IVF outcomes differed according to the pandemic period. The core findings of this study are the significant environment and COVID-19 period interaction effects observed exclusively in the 365-day exposure window for O_3_, CO, and NO_2_ on the mature oocyte rate. The lack of interaction during short-term exposure suggests that acute environmental changes occurring rapidly during lockdown did not immediately affect rapid-maturing follicles. The 365-day window captures the environmental influences on primordial and primary follicle pools, which take months to years to mature into preovulatory follicles. In addition, the chronic pollutant burden may alter the biological susceptibility of ovarian tissue to external stressors, such as those experienced during the pandemic (38, 39).

A notable trend was the directional reversal of pollutant effects across periods, raising important questions about how temporal environmental and societal changes during the pandemic may have modified biological responses. Long-term air quality interacts with broad societal conditions, such as stress and other varying factors, and may alter baseline individual vascular, inflammatory, or endocrine setpoints (40–42). NO_2_ and CO, representative vehicular traffic-related pollutants, induce oxidative stress, systemic inflammation, and endothelial dysfunction, which can impair folliculogenesis, reduce oocyte competence, and disrupt the cumulus-oocyte complex function (39). *In vivo* and *in vitro* studies have consistently demonstrated that long-term exposure to combustion-related pollutants accelerates follicular atresia, impairs cumulus-oocyte complex function, reduces mitochondrial integrity, and alters steroidogenesis, making oocytes more vulnerable to secondary physiological stressors (14, 43–45). Another environmental factor, O_3_, decays rapidly indoors via surface deposition, and indoor concentrations often fall to 10%–30% of outdoor levels (46, 47). Therefore, ambient O_3_ concentration should not be interpreted as direct measures of individual level exposure.

Our findings expand upon the current understanding of environmental reproductive epidemiology, which assumes a static linear relationship between pollutant concentrations and health outcomes. The significant interaction effects demonstrate that the associations between environmental exposure and reproductive outcomes may vary depending on the broader societal context (48). During major societal disruptions, such as pandemics, the health impacts of environmental exposure may be amplified or altered (40, 49). All environmental, psychological, and physiological stressors interacted synergistically for reproductive health. Specifically, the influence of chronic exposure suggests that a longitudinal perspective is required when assessing environmental risks on reproductive outcomes. Clinicians may consider air quality monitoring, protective behaviors, and stress reduction strategies as part of comprehensive preconception care (50).

A major strength of this study is its large, single tertiary center cohort with standardized clinical and laboratory protocols and the use of national meteorological big data. Furthermore, we used comprehensive biologically relevant time windows, including 365-day chronic exposure, to determine the influence of environmental factors on the different stages of folliculogenesis. Seoul maintained consistent healthcare accessibility throughout the COVID-19 pandemic, and changes more precisely reflected societal and environmental shifts rather than clinical practice disruption.

However, this study has some limitations. First, ambient monitoring station data served as a surrogate for individual-level environmental exposure, which could have led to exposure misclassification. These data do not directly capture personal exposure and should therefore be interpreted as reflecting area-level ambient environmental conditions. Second, individual level COVID-19 infection or vaccination history, as well as unmeasured confounders, such as detailed lifestyle changes, dietary modifications, or psychological stress, were not included in this study. Finally, the study cohort included not only infertile women but also patients with cancer who underwent fertility preservation and those undergoing social freezing.

The COVID-19 pandemic was associated with decreased mature oocyte rates and improved fertilization efficiency, while clinical pregnancy rates remained stable. Long-term exposure to specific gaseous pollutants (CO, NO_2_, and O_3_) showed significant interaction terms with COVID-19 period in relation to oocyte maturation, suggesting that chronic air conditions may shape ovarian vulnerability to societal disruption. However, the observational nature of this study precludes definitive causal inferences. These findings highlight the importance of integrating environmental exposure and contextual public health events when evaluating reproductive outcomes and underscore the need for future studies incorporating personalized exposure assessment and mechanistic biomarkers.

## Data Availability

The datasets generated and/or analyzed during the current study are not publicly available due to institutional and ethical restrictions related to the use of patient-level clinical data, but de-identified data may be made available from the corresponding author upon reasonable request and with appropriate institutional approval.
