# Supplementary material for: Changes in ambient environmental conditions correspond to variations in in vitro fertilization outcomes during the COVID-19 pandemic
Source: Front Endocrinol (Lausanne). 2026 Jun 9;17:1796050. doi: 10.3389/fendo.2026.1796050 (PMC13286806; doi:10.3389/fendo.2026.1796050)
Supplement: Supplementary file 1 [file Table1.docx]

**Supplementary Table 1. Binary logistic regression results: Clinical pregnancy outcomes with COVID-19 interaction**

**Supplementary Table 1A. Binary logistic regression: p-values for model selection**

| Variable | Period | N | p-value | | | | Indication | Protocol | Fert. method | TMSC |
| --- | --- | --- | --- | --- | --- | --- | --- | --- | --- | --- |
|  |  |  | Environment | Age | COVID | Env × COVID |  |  |  |  |
| Period 1 | | | | | | |  |  |  |  |
| CO | Period 1 | 662 | 0.071 | < 0.001*** | 0.219 | 0.849 | 0.201 | 0.204 | 0.075 | 0.532 |
| Humidity | Period 1 | 662 | 0.183 | < 0.001*** | 0.272 | 0.372 | 0.205 | 0.223 | 0.048* | 0.397 |
| NO₂ | Period 1 | 662 | 0.010* | < 0.001*** | 0.153 | 0.303 | 0.189 | 0.22 | 0.080 | 0.569 |
| O₃ | Period 1 | 662 | 0.572 | < 0.001*** | 0.32 | 0.788 | 0.182 | 0.225 | 0.066 | 0.44 |
| PM₁₀ | Period 1 | 662 | 0.096 | < 0.001*** | 0.284 | 0.361 | 0.178 | 0.196 | 0.042* | 0.483 |
| PM₂.₅ | Period 1 | 662 | 0.036* | < 0.001*** | 0.237 | 0.135 | 0.198 | 0.181 | 0.094 | 0.566 |
| SO₂ | Period 1 | 662 | 0.088 | < 0.001*** | 0.144 | 0.165 | 0.183 | 0.211 | 0.085 | 0.56 |
| Temperature | Period 1 | 662 | 0.389 | < 0.001*** | 0.29 | 0.357 | 0.193 | 0.24 | 0.058 | 0.448 |
| Period 2 | | | | | | |  |  |  |  |
| CO | Period 2 | 662 | 0.158 | < 0.001*** | 0.281 | 0.901 | 0.211 | 0.197 | 0.048* | 0.467 |
| Humidity | Period 2 | 662 | 0.162 | < 0.001*** | 0.3 | 0.218 | 0.181 | 0.199 | 0.049* | 0.34 |
| NO₂ | Period 2 | 662 | 0.312 | < 0.001*** | 0.318 | 0.641 | 0.184 | 0.206 | 0.051 | 0.476 |
| O₃ | Period 2 | 662 | 0.878 | < 0.001*** | 0.327 | 0.955 | 0.188 | 0.199 | 0.052 | 0.431 |
| PM₁₀ | Period 2 | 662 | 0.007** | < 0.001*** | 0.287 | 0.258 | 0.21 | 0.253 | 0.048* | 0.411 |
| PM₂.₅ | Period 2 | 662 | 0.037* | < 0.001*** | 0.254 | 0.469 | 0.199 | 0.223 | 0.052 | 0.437 |
| SO₂ | Period 2 | 662 | 0.743 | < 0.001*** | 0.314 | 0.518 | 0.181 | 0.208 | 0.058 | 0.453 |
| Temperature | Period 2 | 662 | 0.730 | < 0.001*** | 0.313 | 0.486 | 0.189 | 0.229 | 0.056 | 0.423 |
| Period 3 | | | | | | |  |  |  |  |
| CO | Period 3 | 662 | 0.880 | < 0.001*** | 0.32 | 0.269 | 0.21 | 0.196 | 0.063 | 0.435 |
| Humidity | Period 3 | 662 | 0.634 | < 0.001*** | 0.31 | 0.040* | 0.173 | 0.246 | 0.046* | 0.402 |
| NO₂ | Period 3 | 662 | 0.398 | < 0.001*** | 0.308 | 0.904 | 0.201 | 0.221 | 0.058 | 0.436 |
| O₃ | Period 3 | 662 | 0.699 | < 0.001*** | 0.311 | 0.707 | 0.185 | 0.2 | 0.051 | 0.408 |
| PM₁₀ | Period 3 | 662 | 0.294 | < 0.001*** | 0.303 | 0.317 | 0.191 | 0.224 | 0.050 | 0.451 |
| PM₂.₅ | Period 3 | 662 | 0.305 | < 0.001*** | 0.288 | 0.844 | 0.191 | 0.228 | 0.057 | 0.441 |
| SO₂ | Period 3 | 662 | 0.566 | < 0.001*** | 0.291 | 0.342 | 0.182 | 0.218 | 0.059 | 0.482 |
| Temperature | Period 3 | 662 | 0.781 | < 0.001*** | 0.315 | 0.614 | 0.185 | 0.219 | 0.059 | 0.432 |
| Period 4 | | | | | | |  |  |  |  |
| CO | Period 4 | 662 | 0.294 | < 0.001*** | 0.264 | 0.944 | 0.212 | 0.216 | 0.059 | 0.466 |
| Humidity | Period 4 | 662 | 0.439 | < 0.001*** | 0.295 | 0.106 | 0.19 | 0.227 | 0.045* | 0.393 |
| NO₂ | Period 4 | 662 | 0.072 | < 0.001*** | 0.213 | 0.633 | 0.202 | 0.239 | 0.066 | 0.508 |
| O₃ | Period 4 | 662 | 0.871 | < 0.001*** | 0.323 | 0.955 | 0.184 | 0.212 | 0.057 | 0.423 |
| PM₁₀ | Period 4 | 662 | 0.084 | < 0.001*** | 0.273 | 0.227 | 0.195 | 0.214 | 0.048* | 0.483 |
| PM₂.₅ | Period 4 | 662 | 0.077 | < 0.001*** | 0.233 | 0.233 | 0.201 | 0.232 | 0.074 | 0.52 |
| SO₂ | Period 4 | 662 | 0.265 | < 0.001*** | 0.218 | 0.163 | 0.184 | 0.227 | 0.074 | 0.525 |
| Temperature | Period 4 | 662 | 0.561 | < 0.001*** | 0.301 | 0.498 | 0.19 | 0.234 | 0.059 | 0.441 |
| 90 days | | | | | | |  |  |  |  |
| CO | 90 days | 662 | 0.393 | < 0.001*** | 0.335 | 0.176 | 0.185 | 0.185 | 0.056 | 0.385 |
| Humidity | 90 days | 662 | 0.283 | < 0.001*** | 0.335 | 0.168 | 0.182 | 0.165 | 0.037* | 0.393 |
| NO₂ | 90 days | 662 | 0.134 | < 0.001*** | 0.282 | 0.049* | 0.181 | 0.18 | 0.046* | 0.423 |
| O₃ | 90 days | 662 | 0.345 | < 0.001*** | 0.363 | 0.210 | 0.183 | 0.208 | 0.057 | 0.36 |
| PM₁₀ | 90 days | 662 | 0.324 | < 0.001*** | 0.353 | 0.155 | 0.189 | 0.151 | 0.039* | 0.375 |
| PM₂.₅ | 90 days | 662 | 0.326 | < 0.001*** | 0.345 | 0.054 | 0.186 | 0.153 | 0.042* | 0.387 |
| SO₂ | 90 days | 662 | 0.483 | < 0.001*** | 0.323 | 0.014* | 0.193 | 0.161 | 0.047* | 0.376 |
| Temperature | 90 days | 662 | 0.170 | < 0.001*** | 0.348 | 0.077 | 0.187 | 0.194 | 0.043* | 0.404 |
| 365-day | | | | | | |  |  |  |  |
| CO | 365 days | 662 | 0.138 | < 0.001*** | 0.176 | 0.404 | 0.22 | 0.16 | 0.033* | 0.316 |
| Humidity | 365 days | 662 | 0.912 | < 0.001*** | 0.38 | 0.250 | 0.201 | 0.166 | 0.052 | 0.36 |
| NO₂ | 365 days | 662 | 0.997 | < 0.001*** | 0.388 | 0.077 | 0.209 | 0.147 | 0.048* | 0.365 |
| O₃ | 365 days | 662 | 0.581 | < 0.001*** | 0.438 | 0.020* | 0.193 | 0.135 | 0.049* | 0.375 |
| PM₁₀ | 365 days | 662 | 0.905 | < 0.001*** | 0.381 | 0.536 | 0.201 | 0.156 | 0.047* | 0.347 |
| PM₂.₅ | 365 days | 662 | 0.650 | < 0.001*** | 0.357 | 0.542 | 0.214 | 0.159 | 0.043* | 0.338 |
| SO₂ | 365 days | 662 | 0.916 | < 0.001*** | 0.379 | 0.170 | 0.196 | 0.159 | 0.051 | 0.376 |
| Temperature | 365 days | 662 | 0.872 | < 0.001*** | 0.377 | 0.013* | 0.215 | 0.176 | 0.047* | 0.377 |

Binary logistic regression model: Pregnancy Success ~ Environmental Variable × COVID Period + Age + Indication + Protocol + Fertilization method + TMSC. Outcome: 0 = Failure (No pregnancy + Biochemical), 1 = Success (Intrauterine pregnancy). All environmental variables are z-score standardized. P-values for environmental, COVID, interaction, and covariate terms are from Likelihood Ratio Tests (LRT).

*p < 0.05, **p < 0.01, ***p < 0.001

**Environment:** Environment, Indication, Protocol, Fert method, TMSC: Likelihood Ratio Test (LRT). Age: individual z-test (per year). COVID: LRT (During + Post). Env × COVID: LRT (interaction terms).

**Supplementary Table 1B. Binary logistic regression coefficients: All variables**

| Variable | Period | Intercept | Environment | COVID Period | | Interaction | | Age | Fert. preservation | PGT | Protocol: Soft | Long agonist | Microflare | Fert: all IVF | Fert: IVF+ICSI | TMSC |
| --- | --- | --- | --- | --- | --- | --- | --- | --- | --- | --- | --- | --- | --- | --- | --- | --- |
|  |  |  |  | During | Post | Env × During | Env × Post |  |  |  |  |  |  |  |  |  |
| Period 1 | | | | | | | | |  |  |  |  |  |  |  |  |
| CO | Period 1 | 4.15*** (2.43, 5.87) | 0.11 (-0.24, 0.47) | 0.27 (-0.26, 0.79) | -0.09 (-0.70, 0.53) | 0.03 (-0.45, 0.50) | 0.13 (-0.33, 0.58) | -0.15*** (-0.19, -0.10) | 0.13 (-0.84, 1.10) | -14.19*** (-15.48, -12.90) | 0.19 (-0.32, 0.69) | -0.66 (-1.68, 0.36) | -0.35 (-0.94, 0.24) | 0.53 (-0.21, 1.27) | 0.44* (0.02, 0.85) | -0.05 (-0.19, 0.09) |
| Humidity | Period 1 | 4.39*** (2.65, 6.13) | 0.03 (-0.38, 0.44) | 0.17 (-0.36, 0.70) | -0.11 (-0.73, 0.51) | -0.13 (-0.63, 0.36) | -0.41 (-0.99, 0.18) | -0.15*** (-0.20, -0.11) | 0.13 (-0.82, 1.09) | -14.18*** (-15.46, -12.91) | 0.22 (-0.28, 0.72) | -0.58 (-1.61, 0.45) | -0.35 (-0.96, 0.26) | 0.55 (-0.20, 1.29) | 0.48* (0.06, 0.89) | -0.06 (-0.21, 0.09) |
| NO₂ | Period 1 | 4.12*** (2.39, 5.86) | 0.19 (-0.20, 0.59) | 0.37 (-0.22, 0.96) | 0.26 (-0.42, 0.93) | 0.01 (-0.49, 0.50) | 0.43 (-0.18, 1.04) | -0.15*** (-0.20, -0.10) | 0.17 (-0.79, 1.14) | -14.21*** (-15.51, -12.92) | 0.18 (-0.32, 0.68) | -0.64 (-1.67, 0.39) | -0.35 (-0.95, 0.25) | 0.53 (-0.22, 1.28) | 0.43* (0.01, 0.85) | -0.04 (-0.18, 0.10) |
| O₃ | Period 1 | 4.35*** (2.61, 6.08) | 0.07 (-0.39, 0.54) | 0.14 (-0.44, 0.72) | -0.17 (-0.83, 0.49) | -0.14 (-0.67, 0.39) | -0.20 (-0.79, 0.38) | -0.15*** (-0.20, -0.10) | 0.12 (-0.84, 1.08) | -14.28*** (-15.58, -12.97) | 0.19 (-0.31, 0.69) | -0.61 (-1.61, 0.40) | -0.36 (-0.95, 0.24) | 0.50 (-0.25, 1.24) | 0.46* (0.04, 0.87) | -0.06 (-0.20, 0.09) |
| PM₁₀ | Period 1 | 4.32*** (2.59, 6.05) | 0.13 (-0.25, 0.51) | 0.20 (-0.28, 0.69) | -0.17 (-0.75, 0.41) | -0.08 (-0.54, 0.38) | 0.23 (-0.25, 0.71) | -0.15*** (-0.20, -0.10) | 0.16 (-0.80, 1.12) | -14.28*** (-15.58, -12.98) | 0.23 (-0.27, 0.73) | -0.63 (-1.66, 0.39) | -0.34 (-0.94, 0.26) | 0.56 (-0.19, 1.31) | 0.49* (0.07, 0.90) | -0.05 (-0.19, 0.09) |
| PM₂.₅ | Period 1 | 4.26*** (2.55, 5.98) | 0.08 (-0.20, 0.36) | 0.23 (-0.26, 0.72) | -0.10 (-0.68, 0.48) | 0.08 (-0.33, 0.48) | 0.55* (0.04, 1.05) | -0.15*** (-0.20, -0.10) | 0.14 (-0.81, 1.09) | -14.20*** (-15.48, -12.93) | 0.19 (-0.31, 0.69) | -0.67 (-1.71, 0.38) | -0.37 (-0.97, 0.24) | 0.51 (-0.24, 1.26) | 0.42 (-0.00, 0.83) | -0.04 (-0.18, 0.10) |
| SO₂ | Period 1 | 4.03*** (2.28, 5.77) | 0.15 (-0.17, 0.48) | 0.41 (-0.21, 1.03) | 0.64 (-0.17, 1.45) | -0.00 (-0.57, 0.56) | 0.89* (0.04, 1.75) | -0.15*** (-0.19, -0.10) | 0.16 (-0.79, 1.10) | -14.25*** (-15.54, -12.96) | 0.16 (-0.34, 0.65) | -0.65 (-1.67, 0.38) | -0.38 (-0.97, 0.22) | 0.48 (-0.28, 1.24) | 0.44* (0.02, 0.85) | -0.04 (-0.19, 0.10) |
| Temperature | Period 1 | 4.30*** (2.58, 6.01) | 0.08 (-0.25, 0.42) | 0.16 (-0.32, 0.65) | -0.21 (-0.79, 0.37) | -0.17 (-0.60, 0.27) | -0.37 (-0.85, 0.11) | -0.15*** (-0.20, -0.10) | 0.15 (-0.80, 1.09) | -14.21*** (-15.50, -12.93) | 0.21 (-0.29, 0.71) | -0.57 (-1.59, 0.44) | -0.34 (-0.94, 0.26) | 0.52 (-0.22, 1.26) | 0.46* (0.05, 0.88) | -0.06 (-0.20, 0.09) |
| Period 2 | | | | | | | | |  |  |  |  |  |  |  |  |
| CO | Period 2 | 4.19*** (2.48, 5.89) | 0.16 (-0.14, 0.47) | 0.28 (-0.24, 0.81) | -0.02 (-0.64, 0.59) | -0.07 (-0.50, 0.36) | 0.04 (-0.43, 0.51) | -0.15*** (-0.20, -0.10) | 0.13 (-0.85, 1.10) | -14.16*** (-15.45, -12.87) | 0.21 (-0.29, 0.71) | -0.65 (-1.66, 0.36) | -0.34 (-0.94, 0.25) | 0.58 (-0.17, 1.32) | 0.47* (0.06, 0.88) | -0.05 (-0.20, 0.09) |
| Humidity | Period 2 | 4.61*** (2.90, 6.32) | 0.44* (0.05, 0.83) | -0.01 (-0.49, 0.47) | -0.35 (-0.94, 0.24) | -0.39 (-0.84, 0.07) | -0.44 (-1.04, 0.15) | -0.15*** (-0.20, -0.11) | 0.12 (-0.84, 1.09) | -14.29*** (-15.63, -12.95) | 0.20 (-0.30, 0.70) | -0.63 (-1.65, 0.39) | -0.36 (-0.96, 0.23) | 0.56 (-0.19, 1.31) | 0.47* (0.06, 0.89) | -0.07 (-0.22, 0.08) |
| NO₂ | Period 2 | 4.18*** (2.47, 5.88) | 0.13 (-0.26, 0.53) | 0.27 (-0.29, 0.83) | 0.04 (-0.61, 0.70) | -0.10 (-0.60, 0.39) | 0.14 (-0.40, 0.69) | -0.15*** (-0.20, -0.10) | 0.14 (-0.82, 1.11) | -14.26*** (-15.55, -12.97) | 0.20 (-0.30, 0.70) | -0.64 (-1.66, 0.37) | -0.35 (-0.94, 0.24) | 0.55 (-0.20, 1.30) | 0.47* (0.06, 0.88) | -0.05 (-0.20, 0.09) |
| O₃ | Period 2 | 4.31*** (2.57, 6.04) | -0.05 (-0.48, 0.39) | 0.20 (-0.35, 0.75) | -0.16 (-0.80, 0.48) | 0.02 (-0.49, 0.52) | 0.08 (-0.47, 0.62) | -0.15*** (-0.20, -0.10) | 0.10 (-0.86, 1.06) | -14.26*** (-15.58, -12.95) | 0.20 (-0.30, 0.70) | -0.61 (-1.62, 0.39) | -0.38 (-0.96, 0.21) | 0.53 (-0.22, 1.27) | 0.47* (0.06, 0.88) | -0.06 (-0.20, 0.09) |
| PM₁₀ | Period 2 | 4.24*** (2.53, 5.95) | 0.10 (-0.25, 0.44) | 0.20 (-0.29, 0.68) | -0.17 (-0.75, 0.42) | 0.10 (-0.30, 0.50) | 0.43 (-0.09, 0.95) | -0.15*** (-0.20, -0.10) | 0.12 (-0.80, 1.04) | -14.16*** (-15.44, -12.89) | 0.17 (-0.34, 0.68) | -0.59 (-1.60, 0.43) | -0.37 (-0.99, 0.25) | 0.56 (-0.19, 1.31) | 0.48* (0.06, 0.89) | -0.06 (-0.21, 0.09) |
| PM₂.₅ | Period 2 | 4.24*** (2.53, 5.94) | 0.19 (-0.07, 0.45) | 0.27 (-0.23, 0.78) | -0.04 (-0.63, 0.56) | -0.07 (-0.45, 0.30) | 0.26 (-0.23, 0.76) | -0.15*** (-0.20, -0.10) | 0.14 (-0.81, 1.08) | -14.20*** (-15.49, -12.90) | 0.18 (-0.31, 0.68) | -0.67 (-1.69, 0.35) | -0.33 (-0.93, 0.28) | 0.58 (-0.18, 1.34) | 0.46* (0.05, 0.87) | -0.06 (-0.20, 0.09) |
| SO₂ | Period 2 | 4.21*** (2.49, 5.93) | 0.06 (-0.28, 0.40) | 0.22 (-0.40, 0.84) | 0.17 (-0.64, 0.98) | -0.15 (-0.71, 0.41) | 0.38 (-0.39, 1.14) | -0.15*** (-0.19, -0.10) | 0.13 (-0.82, 1.09) | -14.27*** (-15.57, -12.98) | 0.19 (-0.31, 0.69) | -0.62 (-1.63, 0.38) | -0.36 (-0.95, 0.22) | 0.51 (-0.23, 1.26) | 0.46* (0.05, 0.88) | -0.06 (-0.20, 0.09) |
| Temperature | Period 2 | 4.33*** (2.61, 6.05) | 0.12 (-0.22, 0.47) | 0.15 (-0.34, 0.64) | -0.21 (-0.78, 0.37) | -0.17 (-0.60, 0.25) | -0.31 (-0.79, 0.18) | -0.15*** (-0.20, -0.10) | 0.14 (-0.82, 1.09) | -14.24*** (-15.52, -12.95) | 0.21 (-0.29, 0.71) | -0.58 (-1.59, 0.43) | -0.35 (-0.94, 0.24) | 0.52 (-0.21, 1.26) | 0.46* (0.05, 0.88) | -0.06 (-0.20, 0.09) |
| Period 3 | | | | | | | | |  |  |  |  |  |  |  |  |
| CO | Period 3 | 4.35*** (2.58, 6.11) | 0.13 (-0.26, 0.53) | 0.29 (-0.27, 0.85) | -0.14 (-0.79, 0.50) | -0.00 (-0.49, 0.49) | -0.37 (-0.85, 0.10) | -0.15*** (-0.20, -0.11) | 0.08 (-0.88, 1.04) | -14.18*** (-15.47, -12.89) | 0.21 (-0.29, 0.71) | -0.63 (-1.62, 0.37) | -0.36 (-0.96, 0.23) | 0.54 (-0.21, 1.28) | 0.45* (0.04, 0.86) | -0.06 (-0.20, 0.09) |
| Humidity | Period 3 | 4.48*** (2.72, 6.24) | 0.13 (-0.33, 0.59) | 0.09 (-0.46, 0.64) | -0.16 (-0.79, 0.46) | -0.09 (-0.60, 0.43) | -0.74* (-1.42, -0.06) | -0.15*** (-0.20, -0.11) | 0.17 (-0.80, 1.14) | -14.30*** (-15.63, -12.98) | 0.20 (-0.29, 0.70) | -0.60 (-1.63, 0.42) | -0.33 (-0.93, 0.27) | 0.53 (-0.22, 1.27) | 0.49* (0.07, 0.90) | -0.06 (-0.21, 0.08) |
| NO₂ | Period 3 | 4.33*** (2.54, 6.13) | 0.01 (-0.44, 0.47) | 0.21 (-0.43, 0.85) | -0.10 (-0.87, 0.66) | 0.12 (-0.41, 0.65) | 0.08 (-0.57, 0.73) | -0.15*** (-0.20, -0.10) | 0.11 (-0.85, 1.07) | -14.20*** (-15.48, -12.92) | 0.21 (-0.29, 0.71) | -0.60 (-1.61, 0.41) | -0.35 (-0.94, 0.24) | 0.52 (-0.22, 1.26) | 0.46* (0.05, 0.88) | -0.06 (-0.20, 0.09) |
| O₃ | Period 3 | 4.39*** (2.64, 6.14) | 0.03 (-0.47, 0.53) | 0.16 (-0.42, 0.74) | -0.27 (-0.96, 0.41) | -0.06 (-0.63, 0.51) | 0.13 (-0.47, 0.74) | -0.15*** (-0.20, -0.11) | 0.10 (-0.86, 1.06) | -14.28*** (-15.61, -12.96) | 0.21 (-0.29, 0.71) | -0.60 (-1.62, 0.41) | -0.37 (-0.96, 0.22) | 0.52 (-0.22, 1.27) | 0.47* (0.06, 0.89) | -0.06 (-0.20, 0.08) |
| PM₁₀ | Period 3 | 4.43*** (2.71, 6.15) | -0.06 (-0.46, 0.33) | 0.16 (-0.33, 0.65) | -0.18 (-0.76, 0.40) | 0.12 (-0.34, 0.59) | 0.39 (-0.15, 0.93) | -0.15*** (-0.20, -0.11) | 0.12 (-0.83, 1.07) | -14.24*** (-15.52, -12.95) | 0.22 (-0.28, 0.72) | -0.60 (-1.63, 0.43) | -0.34 (-0.94, 0.26) | 0.52 (-0.22, 1.27) | 0.48* (0.06, 0.89) | -0.06 (-0.20, 0.09) |
| PM₂.₅ | Period 3 | 4.31*** (2.59, 6.03) | 0.04 (-0.27, 0.35) | 0.22 (-0.30, 0.74) | -0.10 (-0.72, 0.51) | 0.09 (-0.33, 0.52) | 0.15 (-0.41, 0.72) | -0.15*** (-0.20, -0.10) | 0.11 (-0.84, 1.06) | -14.24*** (-15.52, -12.96) | 0.21 (-0.29, 0.71) | -0.60 (-1.62, 0.42) | -0.34 (-0.94, 0.26) | 0.53 (-0.22, 1.27) | 0.46* (0.05, 0.88) | -0.06 (-0.20, 0.09) |
| SO₂ | Period 3 | 4.22*** (2.44, 5.99) | 0.07 (-0.26, 0.40) | 0.25 (-0.40, 0.89) | 0.48 (-0.52, 1.47) | -0.12 (-0.70, 0.46) | 0.78 (-0.37, 1.94) | -0.15*** (-0.20, -0.10) | 0.13 (-0.81, 1.07) | -14.27*** (-15.56, -12.98) | 0.18 (-0.32, 0.67) | -0.63 (-1.63, 0.37) | -0.37 (-0.96, 0.23) | 0.50 (-0.25, 1.26) | 0.47* (0.05, 0.88) | -0.05 (-0.20, 0.09) |
| Temperature | Period 3 | 4.32*** (2.60, 6.04) | 0.10 (-0.26, 0.47) | 0.15 (-0.34, 0.64) | -0.20 (-0.79, 0.38) | -0.13 (-0.57, 0.31) | -0.26 (-0.76, 0.24) | -0.15*** (-0.20, -0.10) | 0.14 (-0.82, 1.10) | -14.26*** (-15.55, -12.96) | 0.21 (-0.30, 0.71) | -0.60 (-1.60, 0.41) | -0.36 (-0.95, 0.23) | 0.52 (-0.23, 1.26) | 0.46* (0.05, 0.88) | -0.06 (-0.20, 0.09) |
| Period 4 | | | | | | | | |  |  |  |  |  |  |  |  |
| CO | Period 4 | 4.24*** (2.49, 5.98) | 0.10 (-0.26, 0.47) | 0.27 (-0.28, 0.83) | -0.09 (-0.72, 0.55) | 0.03 (-0.44, 0.51) | -0.05 (-0.52, 0.43) | -0.15*** (-0.20, -0.10) | 0.11 (-0.86, 1.08) | -14.16*** (-15.45, -12.87) | 0.21 (-0.29, 0.71) | -0.61 (-1.62, 0.39) | -0.34 (-0.94, 0.25) | 0.54 (-0.20, 1.29) | 0.46* (0.04, 0.87) | -0.05 (-0.20, 0.09) |
| Humidity | Period 4 | 4.49*** (2.73, 6.26) | 0.12 (-0.33, 0.57) | 0.10 (-0.45, 0.65) | -0.14 (-0.77, 0.49) | -0.14 (-0.66, 0.38) | -0.66 (-1.35, 0.02) | -0.15*** (-0.20, -0.11) | 0.16 (-0.80, 1.12) | -14.23*** (-15.54, -12.92) | 0.22 (-0.28, 0.73) | -0.60 (-1.63, 0.44) | -0.33 (-0.94, 0.27) | 0.54 (-0.21, 1.28) | 0.49* (0.07, 0.90) | -0.06 (-0.21, 0.09) |
| NO₂ | Period 4 | 4.19*** (2.41, 5.96) | 0.10 (-0.36, 0.56) | 0.30 (-0.35, 0.95) | 0.14 (-0.61, 0.90) | 0.08 (-0.47, 0.62) | 0.32 (-0.35, 0.98) | -0.15*** (-0.20, -0.10) | 0.15 (-0.82, 1.11) | -14.18*** (-15.46, -12.90) | 0.20 (-0.30, 0.70) | -0.61 (-1.62, 0.41) | -0.33 (-0.93, 0.26) | 0.52 (-0.22, 1.27) | 0.45* (0.04, 0.86) | -0.05 (-0.19, 0.09) |
| O₃ | Period 4 | 4.35*** (2.60, 6.10) | 0.02 (-0.46, 0.51) | 0.17 (-0.43, 0.76) | -0.19 (-0.87, 0.49) | -0.07 (-0.62, 0.48) | -0.02 (-0.61, 0.58) | -0.15*** (-0.20, -0.10) | 0.10 (-0.85, 1.06) | -14.28*** (-15.59, -12.96) | 0.20 (-0.30, 0.70) | -0.61 (-1.61, 0.40) | -0.36 (-0.95, 0.23) | 0.52 (-0.23, 1.26) | 0.47* (0.05, 0.88) | -0.06 (-0.20, 0.08) |
| PM₁₀ | Period 4 | 4.42*** (2.69, 6.14) | -0.00 (-0.37, 0.36) | 0.18 (-0.31, 0.67) | -0.19 (-0.77, 0.39) | 0.11 (-0.34, 0.56) | 0.42 (-0.08, 0.93) | -0.15*** (-0.20, -0.11) | 0.14 (-0.81, 1.10) | -14.21*** (-15.49, -12.93) | 0.23 (-0.27, 0.73) | -0.61 (-1.65, 0.42) | -0.33 (-0.93, 0.27) | 0.54 (-0.21, 1.29) | 0.48* (0.07, 0.90) | -0.05 (-0.20, 0.09) |
| PM₂.₅ | Period 4 | 4.29*** (2.58, 6.01) | 0.04 (-0.26, 0.34) | 0.23 (-0.28, 0.74) | -0.06 (-0.66, 0.54) | 0.13 (-0.29, 0.55) | 0.51 (-0.05, 1.06) | -0.15*** (-0.20, -0.10) | 0.13 (-0.81, 1.07) | -14.19*** (-15.47, -12.92) | 0.20 (-0.30, 0.70) | -0.62 (-1.64, 0.41) | -0.34 (-0.94, 0.27) | 0.52 (-0.23, 1.26) | 0.44* (0.03, 0.86) | -0.05 (-0.19, 0.09) |
| SO₂ | Period 4 | 4.11*** (2.35, 5.87) | 0.10 (-0.23, 0.43) | 0.33 (-0.32, 0.97) | 0.72 (-0.22, 1.66) | -0.03 (-0.62, 0.56) | 1.08* (0.04, 2.13) | -0.15*** (-0.19, -0.10) | 0.15 (-0.79, 1.09) | -14.25*** (-15.54, -12.96) | 0.16 (-0.33, 0.65) | -0.64 (-1.65, 0.37) | -0.36 (-0.96, 0.23) | 0.48 (-0.27, 1.24) | 0.45* (0.03, 0.86) | -0.05 (-0.19, 0.10) |
| Temperature | Period 4 | 4.30*** (2.58, 6.01) | 0.09 (-0.27, 0.44) | 0.16 (-0.33, 0.65) | -0.21 (-0.79, 0.38) | -0.14 (-0.58, 0.30) | -0.31 (-0.79, 0.18) | -0.15*** (-0.20, -0.10) | 0.14 (-0.81, 1.10) | -14.23*** (-15.52, -12.94) | 0.21 (-0.29, 0.71) | -0.59 (-1.60, 0.42) | -0.35 (-0.94, 0.25) | 0.52 (-0.22, 1.26) | 0.46* (0.05, 0.87) | -0.06 (-0.20, 0.09) |
| 90 days | | | | | | | | |  |  |  |  |  |  |  |  |
| CO | 90 days | 4.53*** (2.78, 6.28) | -0.10 (-0.45, 0.25) | 0.12 (-0.41, 0.64) | -0.17 (-0.78, 0.44) | 0.15 (-0.31, 0.62) | 0.52* (0.00, 1.03) | -0.15*** (-0.20, -0.11) | 0.14 (-0.81, 1.08) | -14.26*** (-15.54, -12.97) | 0.25 (-0.25, 0.75) | -0.59 (-1.62, 0.43) | -0.35 (-0.96, 0.25) | 0.59 (-0.15, 1.33) | 0.45* (0.04, 0.87) | -0.06 (-0.21, 0.08) |
| Humidity | 90 days | 4.42*** (2.64, 6.20) | -0.09 (-0.55, 0.38) | 0.24 (-0.38, 0.85) | 0.02 (-0.68, 0.71) | 0.09 (-0.46, 0.64) | -0.44 (-1.08, 0.21) | -0.16*** (-0.20, -0.11) | 0.10 (-0.82, 1.03) | -14.29*** (-15.59, -12.99) | 0.26 (-0.24, 0.75) | -0.62 (-1.64, 0.41) | -0.37 (-0.97, 0.24) | 0.61 (-0.14, 1.36) | 0.49* (0.07, 0.91) | -0.06 (-0.21, 0.08) |
| NO₂ | 90 days | 4.47*** (2.68, 6.27) | 0.01 (-0.40, 0.42) | 0.18 (-0.45, 0.80) | 0.14 (-0.57, 0.84) | 0.04 (-0.49, 0.58) | 0.78* (0.11, 1.46) | -0.16*** (-0.20, -0.11) | 0.15 (-0.78, 1.07) | -14.27*** (-15.56, -12.98) | 0.25 (-0.25, 0.75) | -0.62 (-1.66, 0.41) | -0.35 (-0.95, 0.26) | 0.60 (-0.15, 1.34) | 0.47* (0.06, 0.89) | -0.06 (-0.21, 0.09) |
| O₃ | 90 days | 4.56*** (2.78, 6.34) | 0.14 (-0.30, 0.59) | 0.07 (-0.51, 0.66) | -0.14 (-0.80, 0.52) | -0.20 (-0.73, 0.33) | -0.53 (-1.13, 0.08) | -0.15*** (-0.20, -0.11) | 0.16 (-0.80, 1.11) | -14.25*** (-15.54, -12.97) | 0.25 (-0.25, 0.75) | -0.58 (-1.60, 0.44) | -0.33 (-0.93, 0.27) | 0.58 (-0.16, 1.32) | 0.46* (0.04, 0.87) | -0.07 (-0.22, 0.08) |
| PM₁₀ | 90 days | 4.55*** (2.80, 6.31) | -0.02 (-0.36, 0.31) | 0.16 (-0.34, 0.65) | -0.21 (-0.80, 0.37) | 0.01 (-0.44, 0.46) | 0.42 (-0.05, 0.90) | -0.16*** (-0.20, -0.11) | 0.08 (-0.84, 1.01) | -14.27*** (-15.57, -12.96) | 0.27 (-0.23, 0.77) | -0.62 (-1.64, 0.41) | -0.38 (-0.98, 0.23) | 0.61 (-0.13, 1.36) | 0.49* (0.07, 0.90) | -0.07 (-0.21, 0.08) |
| PM₂.₅ | 90 days | 4.62*** (2.85, 6.39) | -0.05 (-0.36, 0.27) | 0.14 (-0.37, 0.65) | -0.15 (-0.74, 0.45) | 0.07 (-0.37, 0.51) | 0.69* (0.12, 1.25) | -0.16*** (-0.21, -0.11) | 0.11 (-0.82, 1.03) | -14.27*** (-15.56, -12.97) | 0.27 (-0.24, 0.77) | -0.62 (-1.65, 0.41) | -0.37 (-0.97, 0.24) | 0.61 (-0.14, 1.35) | 0.48* (0.07, 0.89) | -0.06 (-0.21, 0.08) |
| SO₂ | 90 days | 4.58*** (2.76, 6.41) | -0.05 (-0.41, 0.30) | 0.11 (-0.56, 0.79) | 0.77 (-0.17, 1.71) | 0.17 (-0.48, 0.82) | 1.87** (0.64, 3.10) | -0.16*** (-0.20, -0.11) | 0.08 (-0.83, 0.99) | -14.26*** (-15.55, -12.96) | 0.24 (-0.26, 0.74) | -0.63 (-1.66, 0.40) | -0.38 (-0.98, 0.23) | 0.60 (-0.15, 1.34) | 0.47* (0.06, 0.89) | -0.07 (-0.21, 0.08) |
| Temperature | 90 days | 4.48*** (2.75, 6.22) | 0.03 (-0.29, 0.35) | 0.16 (-0.33, 0.65) | -0.24 (-0.83, 0.35) | -0.09 (-0.52, 0.35) | -0.58* (-1.10, -0.06) | -0.16*** (-0.20, -0.11) | 0.15 (-0.78, 1.08) | -14.24*** (-15.53, -12.96) | 0.26 (-0.24, 0.76) | -0.59 (-1.62, 0.44) | -0.33 (-0.94, 0.27) | 0.60 (-0.14, 1.34) | 0.48* (0.06, 0.89) | -0.06 (-0.21, 0.08) |
| 365 days | | | | | | | | |  |  |  |  |  |  |  |  |
| CO | 365 days | 4.88*** (3.08, 6.67) | -0.20 (-0.57, 0.17) | -0.16 (-0.86, 0.55) | -1.57 (-3.45, 0.32) | -0.01 (-0.78, 0.76) | -1.25 (-3.39, 0.89) | -0.16*** (-0.20, -0.11) | 0.11 (-0.83, 1.05) | -14.13*** (-15.45, -12.80) | 0.25 (-0.25, 0.76) | -0.62 (-1.63, 0.38) | -0.37 (-0.98, 0.23) | 0.68 (-0.06, 1.43) | 0.48* (0.07, 0.89) | -0.07 (-0.22, 0.07) |
| Humidity | 365 days | 4.54*** (2.44, 6.64) | 0.01 (-0.73, 0.76) | 0.14 (-1.14, 1.42) | 0.64 (-1.01, 2.28) | 0.09 (-0.82, 0.99) | -1.09 (-2.69, 0.50) | -0.16*** (-0.20, -0.11) | 0.09 (-0.85, 1.03) | -14.22*** (-15.56, -12.88) | 0.23 (-0.27, 0.73) | -0.65 (-1.66, 0.36) | -0.37 (-0.97, 0.22) | 0.61 (-0.13, 1.35) | 0.45* (0.04, 0.87) | -0.07 (-0.21, 0.08) |
| NO₂ | 365 days | 4.70*** (2.70, 6.71) | -0.13 (-0.77, 0.51) | -0.04 (-1.13, 1.06) | 1.51 (-0.48, 3.50) | -0.00 (-0.98, 0.97) | 2.23* (0.22, 4.24) | -0.16*** (-0.20, -0.11) | 0.06 (-0.86, 0.98) | -14.20*** (-15.53, -12.86) | 0.24 (-0.27, 0.74) | -0.66 (-1.66, 0.34) | -0.38 (-0.98, 0.21) | 0.63 (-0.12, 1.37) | 0.46* (0.05, 0.88) | -0.07 (-0.21, 0.08) |
| O₃ | 365 days | 4.58*** (2.75, 6.41) | 0.12 (-0.32, 0.56) | 0.01 (-0.70, 0.72) | 1.23 (-0.14, 2.59) | -0.18 (-0.81, 0.45) | -1.90* (-3.40, -0.41) | -0.15*** (-0.20, -0.11) | 0.04 (-0.87, 0.95) | -14.27*** (-15.57, -12.96) | 0.25 (-0.25, 0.75) | -0.64 (-1.64, 0.36) | -0.41 (-1.00, 0.19) | 0.60 (-0.14, 1.35) | 0.47* (0.05, 0.88) | -0.07 (-0.21, 0.08) |
| PM₁₀ | 365 days | 4.52*** (2.69, 6.34) | -0.05 (-0.41, 0.31) | 0.08 (-0.63, 0.79) | -0.07 (-0.86, 0.73) | -0.00 (-0.74, 0.73) | 0.42 (-0.32, 1.16) | -0.15*** (-0.20, -0.11) | 0.08 (-0.87, 1.02) | -14.23*** (-15.56, -12.90) | 0.25 (-0.25, 0.75) | -0.63 (-1.63, 0.38) | -0.37 (-0.96, 0.22) | 0.62 (-0.13, 1.37) | 0.46* (0.05, 0.88) | -0.07 (-0.21, 0.07) |
| PM₂.₅ | 365 days | 4.71*** (2.85, 6.57) | -0.14 (-0.58, 0.30) | -0.09 (-0.94, 0.76) | 0.11 (-1.17, 1.39) | 0.01 (-0.97, 0.98) | 0.85 (-0.55, 2.25) | -0.15*** (-0.20, -0.11) | 0.09 (-0.86, 1.04) | -14.17*** (-15.50, -12.83) | 0.26 (-0.24, 0.76) | -0.62 (-1.62, 0.38) | -0.37 (-0.96, 0.22) | 0.65 (-0.09, 1.40) | 0.46* (0.05, 0.88) | -0.07 (-0.21, 0.07) |
| SO₂ | 365 days | 4.43*** (2.51, 6.36) | -0.00 (-0.50, 0.50) | 0.15 (-0.82, 1.11) | 2.08 (-0.34, 4.51) | -0.11 (-1.09, 0.87) | 2.95 (-0.04, 5.94) | -0.15*** (-0.20, -0.11) | 0.08 (-0.87, 1.02) | -14.24*** (-15.57, -12.92) | 0.23 (-0.27, 0.73) | -0.65 (-1.66, 0.36) | -0.38 (-0.97, 0.22) | 0.61 (-0.14, 1.35) | 0.46* (0.04, 0.87) | -0.07 (-0.21, 0.08) |
| Temperature | 365 days | 4.50*** (2.78, 6.22) | 0.01 (-0.17, 0.18) | 0.38 (-0.16, 0.91) | 0.90 (-0.12, 1.92) | -2.10 (-4.66, 0.46) | -3.70* (-6.89, -0.52) | -0.16*** (-0.20, -0.11) | 0.13 (-0.80, 1.05) | -14.13*** (-15.47, -12.79) | 0.22 (-0.29, 0.73) | -0.66 (-1.68, 0.36) | -0.36 (-0.95, 0.24) | 0.63 (-0.12, 1.38) | 0.46* (0.05, 0.88) | -0.06 (-0.21, 0.08) |

Regression coefficients (β) with 95% confidence intervals in parentheses. Odds ratios [OR] are shown in brackets for binary predictors where applicable. For overall significance, refer to LRT p-values in Table 5A. TMSC was rescaled to per 100 million motile sperm (effect per 100 million units).

**Interpretation:**

• Intercept: Baseline log-odds of pregnancy success pre-COVID.

• Environment: Change in log-odds per 1 SD increase pre-COVID. [OR] shows multiplicative effect.

• During/Post: Change in baseline pregnancy success compared to pre-COVID.

• Env × During/Post: Modification of environmental effect. Total effect = Main effect OR × Interaction OR.

• Age: Effect of maternal age per year on pregnancy success.

**Supplementary Table 1C. Significant interactions: Environmental effects modified by COVID-19 period**

| Variable | Period | LRT p | Intercept | Environment | COVID Period | | Interaction | | Age | Fert. preservation | PGT | Protocol: Soft | Long agonist | Microflare | Fert: all IVF | Fert: IVF+ICSI | TMSC |
| --- | --- | --- | --- | --- | --- | --- | --- | --- | --- | --- | --- | --- | --- | --- | --- | --- | --- |
|  |  |  |  |  | During | Post | Env × During | Env × Post |  |  |  |  |  |  |  |  |  |
| SO₂ | 90 days | 0.014* | 4.58*** | –0.041 [0.96] | 0.11 (0.35) | 0.77 (0.48) | 0.17 (0.33) | 1.87** (0.63) | –0.148 [0.86] | 0.08 (-0.83, 0.99) | -14.26*** (-15.55, -12.96) | 0.24 (-0.26, 0.74) | -0.63 (-1.66, 0.40) | -0.38 (-0.98, 0.23) | 0.60 (-0.15, 1.34) | 0.47* (0.06, 0.89) | -0.07 (-0.21, 0.08) |
| Temperature | 365 days | 0.013* | 4.500 | 0.01 [1.01] | 0.383 (0.273) | 0.896 (0.521) | -2.10 [0.12] | -3.70* [0.02] | -0.155 [0.86] | 0.13 (-0.80, 1.05) | -14.13*** (-15.47, -12.79) | 0.22 (-0.29, 0.73) | -0.66 (-1.68, 0.36) | -0.36 (-0.95, 0.24) | 0.63 (-0.12, 1.38) | 0.46* (0.05, 0.88) | -0.06 (-0.21, 0.08) |
| O₃ | 365 days | 0.020* | 4.583 | 0.12 [1.13] | 0.010 (0.364) | 1.226 (0.697) | -0.18 [0.84] | -1.90* [0.15] | -0.155 [0.86] | 0.04 (-0.87, 0.95) | -14.27*** (-15.57, -12.96) | 0.25 (-0.25, 0.75) | -0.64 (-1.64, 0.36) | -0.41 (-1.00, 0.19) | 0.60 (-0.14, 1.35) | 0.47* (0.05, 0.88) | -0.07 (-0.21, 0.08) |
| Humidity | Period 3 | 0.040* | 4.477 | 0.13 [1.14] | 0.092 (0.281) | -0.164 (0.321) | -0.09 [0.91] | -0.74* [0.48] | -0.155 [0.86] | 0.17 (-0.80, 1.14) | -14.30*** (-15.63, -12.98) | 0.20 (-0.29, 0.70) | -0.60 (-1.63, 0.42) | -0.33 (-0.93, 0.27) | 0.53 (-0.22, 1.27) | 0.49* (0.07, 0.90) | -0.06 (-0.21, 0.08) |
| NO₂ | 90 days | 0.049* | 4.474 | 0.01 [1.01] | 0.176 (0.318) | 0.138 (0.359) | 0.04 [1.04] | 0.78* [2.19] | -0.156 [0.86] | 0.15 (-0.78, 1.07) | -14.27*** (-15.56, -12.98) | 0.25 (-0.25, 0.75) | -0.62 (-1.66, 0.41) | -0.35 (-0.95, 0.26) | 0.60 (-0.15, 1.34) | 0.47* (0.06, 0.89) | -0.06 (-0.21, 0.09) |

Variables with a significant Env × COVID interaction (Likelihood Ratio Test p < 0.05 from **Supplementary Table 1**A). These results indicate that the environmental effect on pregnancy success differs significantly across COVID periods.

**Interpretation of Relative Effects:**

• Main Effect OR: Baseline environmental effect during pre-COVID (reference).

• Interaction OR < 1: Environmental effect is weakened in that period.

• Interaction OR > 1: Environmental effect is strengthened in that period.

• Total environmental OR = Main effect OR × Interaction OR.

Model Specification

Model: logit(P(Success)) = β₀ + β₁(Env) + β₂(During) + β₃(Post) + β₄(Env × During) + β₅(Env × Post) + β₆(Age) + β₇(Indication) + β₈(Protocol) + β₉(Fert method) + β₁₀(TMSC)

Outcome Definition:

• 0 (Failure): No pregnancy + Biochemical pregnancy

• 1 (Success): Intrauterine pregnancy

**Exposure Periods:**

• Period 1: Follicular phase (menstruation to trigger day)

• Period 2: Ovulation to embryo transfer

• Period 3: Embryo development (OPU to transfer)

• Period 4: Implantation window (transfer to pregnancy test)

• 90 days: 90-day average exposure before oocyte retrieval

• 365 days: 365-day average exposure before oocyte retrieval

**Abbreviations:** OR, odds ratio; OPU, oocyte pick-up; PM_2.5_, fine particulate matter ≤ 2.5 μm; PM10, particulate matter ≤ 10 μm; SD, standard deviation; SE, standard error; CO, carbon monoxide; COVID-19, coronavirus disease 2019; NO_2_, nitrogen dioxide; O_3_, ozone; SO_2_, sulfur dioxide
